# Glyoxylate protects against cyanide toxicity through metabolic modulation

**DOI:** 10.1038/s41598-022-08803-y

**Published:** 2022-03-23

**Authors:** Jason R. Nielson, Anjali K. Nath, Kim P. Doane, Xu Shi, Jangwoen Lee, Emily G. Tippetts, Kusumika Saha, Jordan Morningstar, Kevin G. Hicks, Adriano Chan, Yanbin Zhao, Amy Kelly, Tara B. Hendry-Hofer, Alyssa Witeof, Patrick Y. Sips, Sari Mahon, Vikhyat S. Bebarta, Vincent Jo Davisson, Gerry R. Boss, Jared Rutter, Calum A. MacRae, Matthew Brenner, Robert E. Gerszten, Randall T. Peterson

**Affiliations:** 1 Department of Pharmacology and Toxicology, College of Pharmacy, University of Utah, Salt Lake City, UT 84112 USA; 2 Department of Cardiology, Beth Israel Deaconess Medical Center, Boston, MA 02115 USA; 3 Broad Institute, Cambridge, MA 02142 USA; 4 Beckman Laser Institute and Department of Medicine, University of California, Irvine, CA 92697 USA; 5 Division of Cardiovascular Medicine, Brigham and Women’s Hospital, Boston, MA 02115 USA; 6 Department of Biochemistry and Howard Hughes Medical Institute, University of Utah, Salt Lake City, USA; 7 Department of Medicine, University of California, San Diego, CA 92093 USA; 8 Department of Emergency Medicine, University of Colorado School of Medicine, Aurora, CO 80045 USA; 9 Department of Biomolecular Medicine, Ghent University, 9000 Ghent, Belgium; 10 Department of Medicinal Chemistry and Molecular Pharmacology, Purdue University, West Lafayette, IN 47907 USA

**Keywords:** Drug discovery, Metabolism, Respiration

## Abstract

Although cyanide’s biological effects are pleiotropic, its most obvious effects are as a metabolic poison. Cyanide potently inhibits cytochrome c oxidase and potentially other metabolic enzymes, thereby unleashing a cascade of metabolic perturbations that are believed to cause lethality. From systematic screens of human metabolites using a zebrafish model of cyanide toxicity, we have identified the TCA-derived small molecule glyoxylate as a potential cyanide countermeasure. Following cyanide exposure, treatment with glyoxylate in both mammalian and non-mammalian animal models confers resistance to cyanide toxicity with greater efficacy and faster kinetics than known cyanide scavengers. Glyoxylate-mediated cyanide resistance is accompanied by rapid pyruvate consumption without an accompanying increase in lactate concentration. Lactate dehydrogenase is required for this effect which distinguishes the mechanism of glyoxylate rescue as distinct from countermeasures based solely on chemical cyanide scavenging. Our metabolic data together support the hypothesis that glyoxylate confers survival at least in part by reversing the cyanide-induced redox imbalances in the cytosol and mitochondria. The data presented herein represent the identification of a potential cyanide countermeasure operating through a novel mechanism of metabolic modulation.

## Introduction

Cyanide poses a risk to human health as an agent in chemical warfare^1^, suicide^2^, occupational exposure^3^, and smoke inhalation^4^. It can be absorbed through the skin, respiratory tract, or mucous membranes, allowing for a variety of modes of entry. Once in the body, cyanide (CN) anions are rapidly distributed throughout the body, affecting the central nervous system first, followed by the cardiovascular and respiratory systems, leading to seizure, dyspnea, arrhythmias, and eventually death.

Cyanide is a metabolic poison, and the symptoms, signs, and objective findings of toxicity are associated with metabolic impairment. Several cyanide countermeasures are available for clinical use, such as sodium nitrite, sodium thiosulfate, dicobalt edetate, hydroxocobalamin and others^5^. These countermeasures, directly or indirectly, function via a stoichiometric reaction with free cyanide to form a less-toxic product. Although this scavenging mechanism of action is effective, there are significant limitations to this approach. First, these countermeasures must be delivered at high enough concentrations to enable reaction with cyanide to reduce it to sub-lethal levels, which typically requires intravenous (i.v.) administration. Furthermore, adequate systemic concentrations of antidote can also produce adverse effects including hypotension, tachycardia, and even steal phenomena^3,6–9^. Scavenging molecules directly target cyanide molecules, and once a cyanide molecule binds to cytochrome c oxidase or another cellular target, scavenging becomes less effective. The identification and development of cyanide countermeasures that do not rely solely on scavenging cyanide, but that can counteract the downstream sequelae of cyanide binding, would be highly valuable for several reasons. An increase in potency that would enable efficacy at sub-stoichiometric levels would reduce the overall dose required. The combination of a novel metabolic modulating agent with existing scavenging agents could potentially lead to more effective countermeasures. Finally, agents that restore metabolic functions could provide benefit even after cyanide binds its cellular target(s).

Cyanide is considered to have multiple cellular targets with the best understood being Complex IV of the mitochondrial electron transport chain (also known as cytochrome c oxidase). By inhibiting Complex IV, cyanide blocks electron flux from cytochrome c to oxygen coupled with pumping of protons across the mitochondrial inner membrane. In most cell types, particularly neurons and cardiomyocytes that are believed to be lethal targets of cyanide in humans, the mitochondrial electron transport chain is required to produce the vast majority of ATP within the cell. Cyanide inhibits Complex IV by binding to the heme a3 moiety, thereby blocking the transfer of electrons to oxygen, stalling the electron transport chain, and preventing mitochondrial ATP production. The inhibition of cytochrome c oxidase, along with potential inhibition of other metabolic enzymes, unleashes a cascade of metabolic perturbations that cause lethality, particularly through effects on excitable cells^1,6^. As such, there is reason to believe that either normalizing these metabolic perturbations or initiating a compensatory response could be an effective strategy for countermeasure development. The feasibility of such an approach is supported by previous work in which metabolic profiling was used to identify differences in energy metabolism in cyanide-resistant vs cyanide-sensitive zebrafish in early embryonic stages. A directed screen of 48 small molecules (all of which had previously been annotated as modulators of energy metabolism) identified modulators of the pyruvate dehydrogenase complex, glyoxylate, and others, as potential cyanide countermeasures^10^.

Here we describe a more comprehensive screen of human metabolites that independently identifies glyoxylate as a potent candidate countermeasure. In addition, we demonstrate that glyoxylate confers cyanide resistance in two established mammalian models of cyanide poisoning. Finally, characterization of the glyoxylate activity in these models suggests additional mechanisms of protection distinct from the cyanide scavenging alone exhibited by existing countermeasures.

## Results

### Systematic screen of human metabolites identifies glyoxylate as a cyanide countermeasure

**Figure 1:**
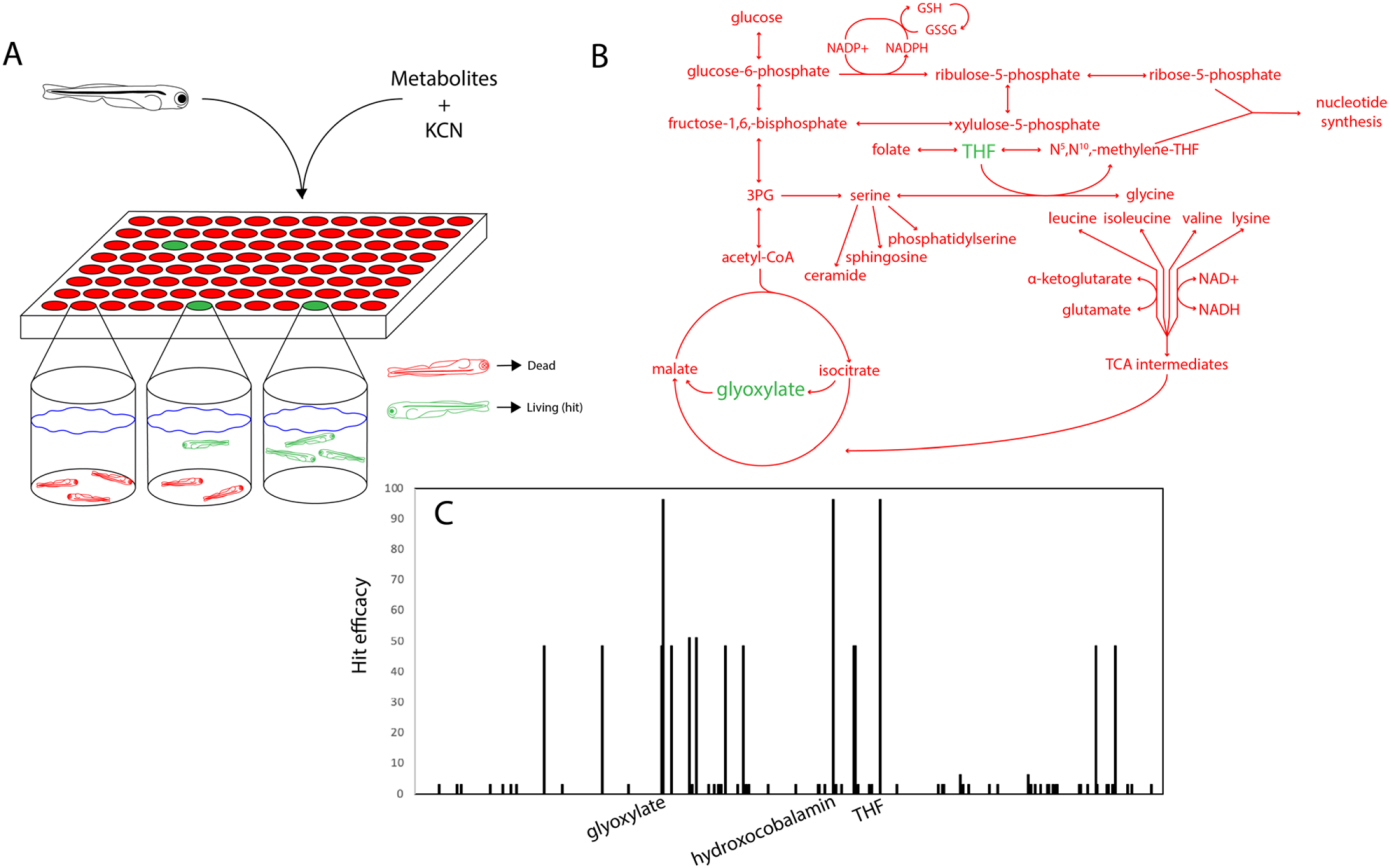
Glyoxylate identified as cyanide antidote in metabolite screen. (**A**) Schematic of the screening process for cyanide toxicity antidotes. 5 dpf zebrafish were added 3 per well to 96 well plates in E3 buffered with 20 mM HEPES and treated with 30 µM human metabolite and 10 µM KCN. (**B**) Metabolic map illustrating the breadth of the screen. Of the ~ 500 human-derived metabolites tested, THF and glyoxylate were the only molecules that yielded 24 h survival in both of the duplicate screens other than hydroxocobalamin, the positive control. (**C**) Hit efficacy plot denoting compound rescue capability. 2 points were assigned for 3 h touch responsiveness and 50 points were assigned for 24 h survival after each of the 2 screens (e.g., a compound that produces 24 h survival in both screens receives a score of 100, one that produces 3 h touch responsiveness in one and 24 h survival in the other receives a score of 52). Each peak represents one compound and its corresponding efficacy score.

Our initial efforts investigating differences in cyanide sensitivity in zebrafish at varying stages of development and identification of potential target pathways for cyanide countermeasures encouraged us to conduct a more comprehensive screen of potential metabolic modulators^10^. This screen included 485 human metabolites, vitamins, and cofactors and was done in an in vivo zebrafish model of cyanide toxicity (Supplementary Table S1). An assay condition was selected that involved zebrafish larvae at 5 days post fertilization (dpf) exposed to a lethal dose of cyanide (10 µM) and individual metabolites from the extended metabolite library at a concentration of 30 µM (Fig. 1A). Rescue of cyanide toxicity was assessed by monitoring responsiveness to touch after 3 h of cyanide exposure and organism survival after 24 h of cyanide exposure. Weighted scores were assigned based on survival (50 points assigned for 24 h survival) and touch responsiveness (2 points assigned for responsiveness to touch at 3 h). Hydroxocobalamin had previously been described as an effective cyanide scavenger^11^ and acted as a positive control for this screen. Two metabolites were identified that reproducibly protected against lethal cyanide exposure in each of the duplicate screens: tetrahydrofolate (THF) and glyoxylate (Fig. 1B,C). Based on literature indicating that enhanced tricarboxylic acid (TCA) cycle activity contributed to the resistance against cyanide in early stage zebrafish embryos^10^ and the ability of alpha-keto acids like glyoxylate to scavenge cyanide via cyanohydrin formation^6^, a more complete investigation of glyoxylate was prioritized.

### Glyoxylate rescues cyanide toxicity in zebrafish, mice, and rabbits

**Figure 2:**
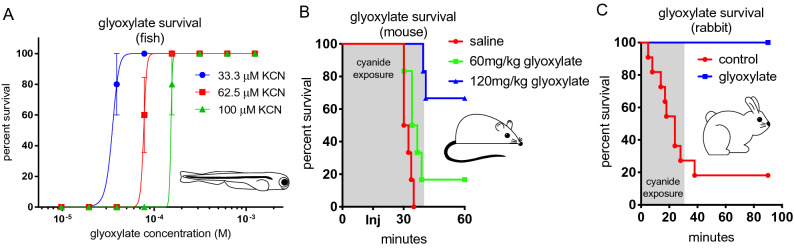
Glyoxylate improves survival rates in vertebrate animal models. (**A**) Percent survival of 5 dpf zebrafish treated with indicated KCN and glyoxylate concentrations, survival assessed 20 h after administration. Fish were arrayed 3 per well. N = 5 wells per glyoxylate concentration in each of the given cyanide conditions. Data are presented as mean ± SEM. (**B**) Mice were exposed for a total of 40 min to 587 ppm HCN gas in a sealed chamber. At 15 min, the mice were removed and received an intramuscular injection of 60 mg/kg glyoxylate (green line), 120 mg/kg glyoxylate (blue line), or saline (red line), and returned to the cyanide chamber for 25 min. N = 6 for all three groups. There was a significant difference in survival between mice injected with 120 mg/kg glyoxylate and mice injected with saline (***p = 0.0005), but not between mice injected with 60 mg/kg glyoxylate and mice injected with saline (p = 0.15). (**C**) An LD80 lethal level dose of i.v. NaCN was administered to rabbits. Upon reaching target systolic BP (50 mm/Hg), 11 control rabbits were injected with 2 mL saline and 10 rabbits were given 2 M glyoxylate while the NaCN infusion continued for 30 min to a total dose of 22 mg. Injection of saline or glyoxylate is designated as time t = 0.

In order to explore the relevance of glyoxylate as a novel antidote with potential clinical applications, we first sought to validate its efficacy in a variety of models. In the zebrafish model, glyoxylate protects against cyanide toxicity in a dose-dependent fashion (Fig. 2A).

To further test and validate the significance of the glyoxylate-mediated rescue of cyanide toxicity observed in zebrafish, we utilized an established pipeline for efficacy testing in complementary mouse and rabbit models^12^. In the mouse model of cyanide inhalation^13^, a simulation more consistent with some real-life cyanide exposure scenarios, intramuscular (i.m.) administered glyoxylate protected against cyanide-induced lethality in 4 of 6 subjects treated with 120 mg/kg glyoxylate over a 60 min time course. In contrast, a 60 mg/kg dose of glyoxylate provided no statistically significant protection over the saline treated animals, all of which expired within 35 min (Fig. 2B). There was a significant difference in survival between mice given 120 mg/kg glyoxylate versus those given saline (***p = 0.0005), but not between mice given 60 mg/kg glyoxylate versus those given saline (p = 0.15). All mice that survived for more than 1 h post cyanide exposure appeared normal and remained clinically normal for two weeks post exposure, at which time they were euthanized. We did not observe any adverse effects of glyoxylate at the doses used. Successful testing in the mouse model justified additional glyoxylate studies in a lethal rabbit model^14^ wherein anesthetized rabbits are infused with i.v. cyanide at a rate of 0.33 mg/min followed by administration of the 2 M glyoxylate countermeasure upon reaching the target blood pressure of 40–58 mmHg systolic (Fig. 2C). Reaching target blood pressure generally took between 5 and 25 min and the cyanide infusion continued 30 min after antidote administration. Impressively, 10 of 10 glyoxylate-treated rabbits survived the cyanide exposure whereas only 2 of 11 control animals survived. Comparing the survival distributions of glyoxylate-treated versus saline (control) groups, the difference is statistically significant (***p < 0.0005).

### Oxygen consumption in cyanide-treated animals is restored with glyoxylate treatment

**Figure 3:**
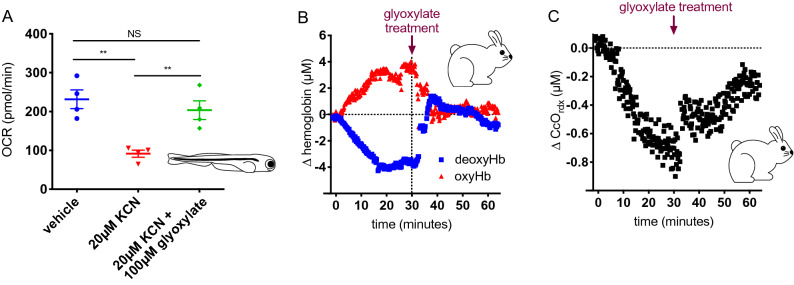
Oxygen metabolism is restored following glyoxylate treatment. (**A**) Oxygen consumption rates of 5 dpf zebrafish treated with DMSO, KCN, or KCN and glyoxylate with the given concentrations (**p < 0.005 using one-way ANOVA). Fish were arrayed 1 per well, N = 4 wells for each condition. (**B**) Glyoxylate was given i.m. to rabbits 30 min after exposure to i.v. cyanide, and deoxygenated and oxygenated hemoglobin were monitored throughout the timecourse with diffuse optical spectroscopy (DOS). Data from a single representative rabbit are shown. (**C**) DOS monitoring of cytochrome C oxidase state. Glyoxylate was administered i.m. 30 min after i.v. cyanide infusion started. Results shown for a single rabbit are typical of the response seen by DOS in animals treated with cyanide and glyoxylate.

In the zebrafish model of cyanide toxicity, 5 dpf embryos were treated with vehicle, cyanide alone, or with a combination of cyanide and glyoxylate. After a 2 h incubation, oxygen consumption was measured. The embryos treated with cyanide alone had severely depressed oxygen consumption rates compared with vehicle treated embryos. Glyoxylate treatment largely restores the oxygen consumption rates in cyanide-treated embryos (Fig. 3A; **p < 0.005). These results are consistent with the restoration of oxygen consumption and aerobic respiration likely through recovery of cytochrome c oxidase function. Oxygen consumption was also measured in these embryos upon treatment with various electron transport chain inhibitors (oligomycin, FCCP, rotenone, and antimycin A) and glyoxylate-mediated rescue was evident under baseline conditions (Supplementary Fig. S1).

Inhibition of cytochrome c oxidase by cyanide prevents normal oxygen metabolism, thereby causing an accumulation of oxygenated hemoglobin and a depletion of deoxygenated hemoglobin^15^. We observed these expected changes in oxygenated and deoxygenated hemoglobin in a rabbit cyanide poisoning model where rabbits are given a sublethal dose of cyanide and spontaneous recovery rates are studied. Furthermore, treatment of these rabbits with glyoxylate led to a rapid recovery of the levels of oxygenated and deoxygenated hemoglobin to baseline levels (Fig. 3B). These same rabbits exhibited an expected reduction in cytochrome c oxidase redox state upon treatment with cyanide that was reversed upon treatment with glyoxylate (Fig. 3C). Previous evidence from these established methods has shown that cytochrome c oxidase redox state and oxygenated/deoxygenated hemoglobin levels do not return to baseline over time with cyanide exposure when a countermeasure is not given^12,15^. These data from both the rabbit and zebrafish models demonstrate that disruption of oxygen consumption caused by cyanide exposure is reversed upon treatment with glyoxylate.

### Glyoxylate forms cyanohydrin species in vitro

An understanding of the mechanism(s) by which glyoxylate provides effective protection against cyanide toxicity in all three animal models, and the restoration of oxygen consumption in two models, could reveal pathways for further development of countermeasures. Initially, we hypothesized that glyoxylate might scavenge cyanide through formation of a cyanohydrin to protect against cyanide toxicity. Alpha-keto acids and related compounds readily form cyanohydrins, with multiple reports providing evidence to suggest that cyanohydrin formation of alpha-keto acids protects against cyanide poisoning^6^. For example, α-ketoglutarate, has been shown to have rescue capability against cyanide poisoning, though its potency is 20-fold less than that of glyoxylate^10^. Glyoxylate, too, is known to form a cyanohydrin, which may thereafter be converted into aldol derivatives^16^. For these reasons, the chemistry of glyoxylate-cyanohydrin under conditions relevant to the use of glyoxylate as a countermeasure was surveyed.

The in vitro incubation of cyanide with glyoxylate resulted in the formation of multiple cyanohydrin adducts detected by quadrupole-time of flight mass spectrometry. To confirm the structures of the newly formed compounds after glyoxylate reacts with cyanide group(s), we began by comparing their mass spectra. Specifically, freshly prepared natural abundance (^12^C^14^N) and isotope-labeled (^13^C^15^N) sodium cyanide solutions (0.135 M, pH 7.4) were prepared. Next, from a stock solution of 20 mM glyoxylate (pH 7.4), glyoxylate was added to the cyanide solution to give a final concentration of 20 µM glyoxylate. After 2 h incubation, their spectra were obtained and compared. As shown in Supplementary Table S2 and Supplementary Fig. S2, common peaks present in both the unaltered and the isotope-labeled samples include *m/z* = 72.9939 and 168.9757. Those m/z values which occur in pairs of 2 mass unit differences represent the natural abundance (^12^C^14^N) and isotope-labeled (^13^C^15^N) versions of the same cyanide addition products to glyoxylate. Any mass pairs related by 4 units represent species with two cyanide adducts to glyoxylate. The most notable observations are summarized in Supplementary Table S2 with the focus on unique peak pairs, including *m/z* = 195.9865/197.9872 and 222.9974/226.9988 observed in each of these samples.

The peak at *m/z* 72.9939 was recognized as unbound glyoxylate monomer. The identification of all other peaks was confirmed through targeted fragmentation. For the base peak at *m/z* 168.9757, the fragment (product) ions only contained glyoxylate, indicating that the corresponding compound is glyoxylate dimer sodium salt. A plausible explanation for the formation of this dimer would involve the reaction of glyoxylate-cyanohydrin with glyoxylate, followed by elimination of cyanide^17^. This pathway is supported by the fragmentation result of the *m/z* 195.99865 species, which produced the *m/z* 168.9759 species via the loss of one cyanide group, the *m/z* 100.0039 species via the loss of glyoxylate, and the *m/z* 72.9936 species (base peak) corresponding to glyoxylate. Therefore, it is hypothesized that the *m/z* 195.986 peak is one of the two major products of cyanide addition to glyoxylate, resulting from the reaction of the glyoxylate-cyanohydrin.

The other major species is at *m/z* 222.9974 and has been identified as a dimer of the glyoxylate-cyanohydrin molecule. Although the formation pathway is unclear, the proposed composition of the *m/z* 222.9974 species is supported by the MS2 fragmentation result; as shown in Supplementary Table S2, the main fragment products were from loss of cyanide (195.9848), loss of two cyanides (168.9751), formation of glyoxylate-cyanohydrin (100.004), and glyoxylate (72.9936). However, the fragmentation pattern also indicated multiple lower-abundance peaks, suggesting further complexity of the ion chemistry of this species (Supplementary Fig. S3). Taken together, these data indicate that glyoxylate can form cyanohydrins by reacting with cyanide in vitro, raising questions about the extent to which cyanohydrin formation contributes to glyoxylate’s in vivo countermeasure activity. We also considered the possibility that glyoxylate might strip cyanide from other cyanohydrins, potentially freeing keto acids such as pyruvate to function again in the TCA cycle. We found some evidence such a reaction can occur between glyoxylate and the pyruvate cyanohydrin in vitro (Supplementary Fig. S4); however, it is unlikely release of pyruvate is a significant contributor to glyoxylate-mediated cyanide rescue in vivo because substantial amounts of pyruvate release would increase overall pyruvate levels, whereas we observe a substantial *decrease* in total pyruvate levels (Fig. 5A). Nevertheless, we have not ruled out the possibility that release of pyruvate makes some small contribution to glyoxylate’s activity.

### Glyoxylate rescues with increased efficacy and kinetics compared to known cyanide-scavenging molecules

**Figure 4:**
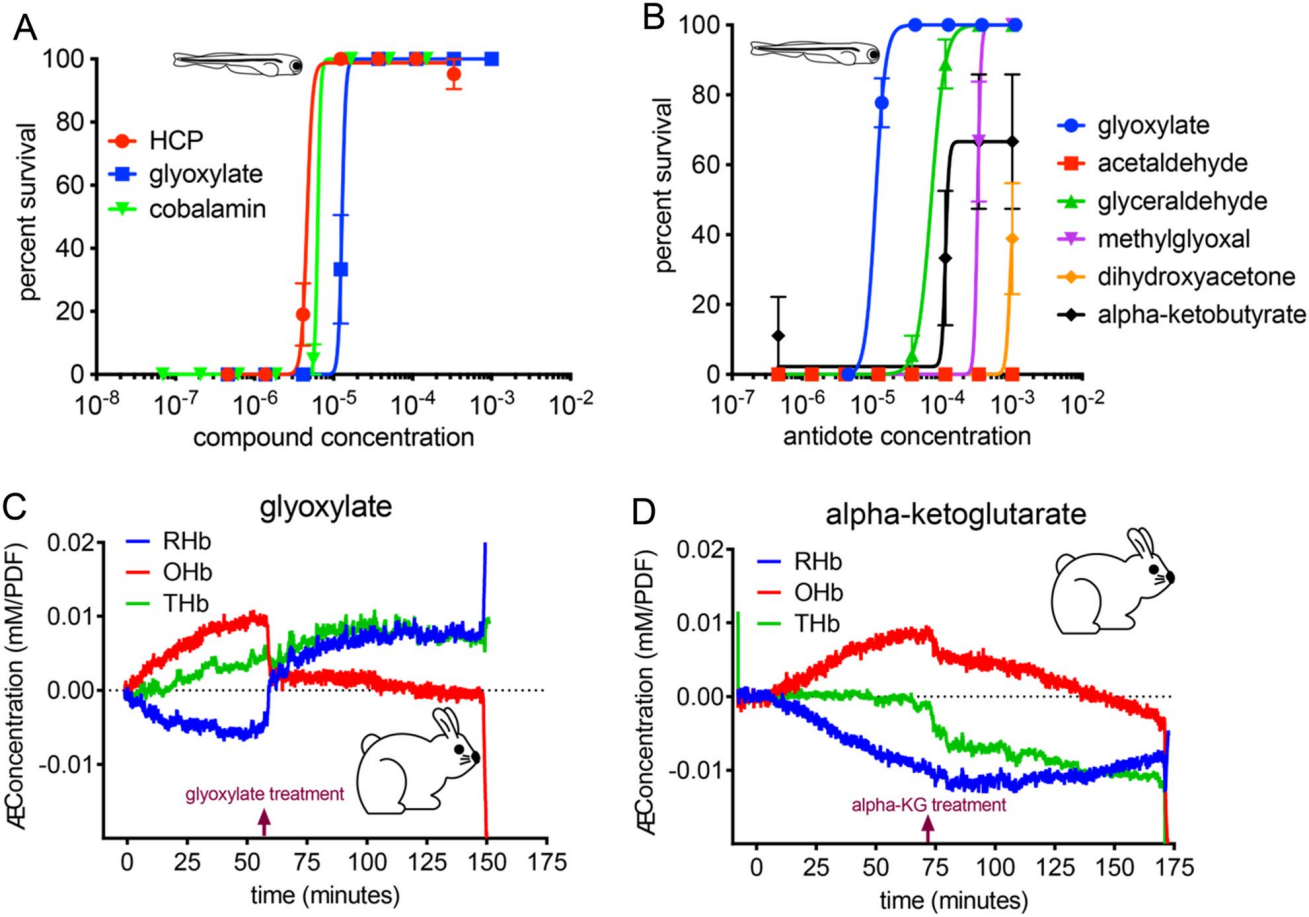
Glyoxylate’s rescue mechanism is not fully explained by cyanohydrin formation. Dose dependent survival of 5 dpf zebrafish in the presence of (**A**) scavengers (3 fish per well, N = 6 wells for each condition) or (**B**) cyanohydrin-forming molecules 20 h after exposure to 20 µM KCN (3 fish per well, N = 6 wells for each condition with the exception of alpha-ketobutyrate, N = 3). Data presented as mean ± SEM. HCP was 100% lethal at the highest dose (1 mM) and this data point was removed to preserve an accurate dose–response curve. (**C**) Continuous-wave near-infrared spectroscopy (CWNIRS) monitoring changes in concentrations of deoxygenated (RHb), oxygenated (OHb), and total (THb) hemoglobin. The rabbits were exposed to a sublethal dose of 10 mg i.v. NaCN for 60 min. At 60 min, subjects received equimolar doses of glyoxylate or (**D**) alpha-ketoglutarate (100 mg or 147 mg respectively). Results shown are typical of the response seen by CWNIRS in animals treated with cyanide and glyoxylate.

Following the observation that glyoxylate forms cyanohydrins in vitro, we compared the efficacy of glyoxylate as a cyanide countermeasure with molecules known to scavenge cyanide by directly binding cyanide (Fig. 4A) or through formation of cyanohydrin adducts (Fig. 4B) in the zebrafish model of cyanide toxicity. Glyoxylate exhibited similar efficacy with known cyanide scavengers, but markedly increased efficacy compared with compounds known to form cyanohydrins. Glyoxylate consistently showed no toxicity throughout the dose range tested unlike hexachloroplatinate (HCP), which was lethal at the highest dose (This data point was excluded for purposes of curve fitting. There was zero percent survival for HCP at 1 mM). Concomitantly, in a sublethal model of cyanide toxicity in rabbits where spontaneous recovery rates are monitored, the expected changes of oxygenated hemoglobin buildup and deoxygenated hemoglobin depletion were seen with cyanide exposure. Treatment of these rabbits with glyoxylate led to a rapid recovery of the levels of oxygenated and deoxygenated hemoglobin to baseline levels (Fig. 4C). This result is in contrast with the response to α-ketoglutarate which showed very gradual return to baseline (Fig. 4D). Comparison of glyoxylate efficacy and rescue kinetics with known cyanohydrin-forming molecules suggests that glyoxylate is working, at least in part, through a mechanism of cyanide rescue that is distinct from cyanohydrin formation.

We next evaluated the efficacy of glyoxylate against two additional metabolic toxins, sodium sulfide and sodium azide, which are known to disrupt normal electron transport chain function. Glyoxylate demonstrated a protective capacity when zebrafish larvae were treated with these two cytochrome c oxidase inhibitors (Supplementary Fig. S5). Evidence of protection against azide and sulfide toxicity, along with glyoxylate’s increased rescue capacity relative to other cyanohydrin donors, suggest that cyanohydrin formation is likely not the only explanation for glyoxylate efficacy. Additionally, protection against azide and sulfide toxicity suggests that the utility of glyoxylate might extend beyond cyanide poisoning. However, we cannot rule out the possibility that sulfide and azide may also be forming adducts with glyoxylate^18–20^, and therefore we cannot rule out scavenging as a contributor to the mechanism of rescue.

### Glyoxylate treatment restores TCA cycle metabolism following cyanide exposure

The efficacy and kinetics of rescue observed in the non-lethal rabbit model compared to other known cyanohydrin-forming metabolites motivated a study of the potential impact of glyoxylate administration on central carbon metabolic pathways under cyanide exposure. To evaluate these effects, mass spectrometry was used to perform metabolite profiling on serum samples from rabbits exposed to non-lethal cyanide and subsequent glyoxylate rescue treatment. Serial blood samples were collected at baseline, during cyanide exposure, and following IM administration of glyoxylate.

**Figure 5:**
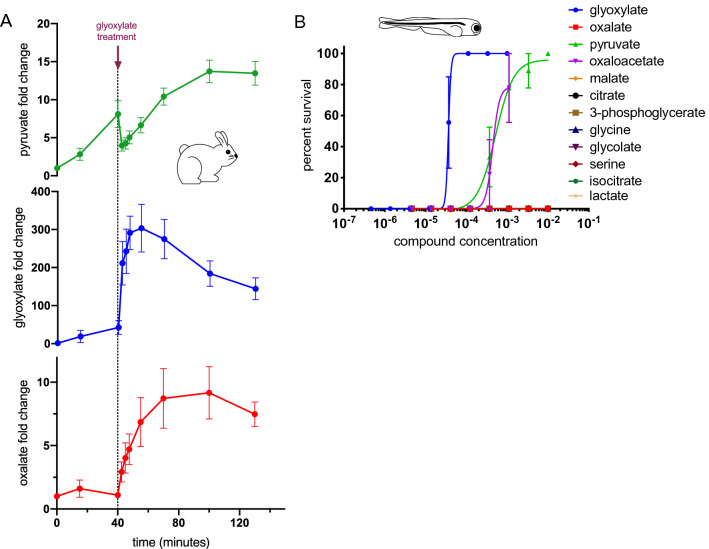
Direct metabolites of glyoxylate do not rescue with comparable efficacy. (**A**) Metabolite profiling was performed on serial plasma samples collected from rabbits at baseline, during cyanide exposure, and subsequent to i.m. administration of glyoxylate. The pharmacokinetic profile of exogenous glyoxylate is depicted in blue. Data are represented as mean fold change from baseline ± SEM, N = 9. (**B**) Dose-dependent survival of 5 dpf zebrafish embryos treated with metabolites closely related to glyoxylate through multiple metabolic pathways. Oxaloacetate was 100% lethal at the two highest doses. These data points were removed to preserve an accurate dose–response curve. 3 fish per well, N = 3 wells. Data presented as mean ± SEM.

Cyanide infusion inhibits cellular respiration and concomitantly slows the consumption of pyruvate and TCA cycle metabolites which leads to increased levels of these metabolites^8^. As expected, we observed a significant increase in pyruvate levels during the 40-min cyanide infusion prior to glyoxylate treatment (Fig. 5A). The administration of glyoxylate resulted in an almost instantaneous consumption of pyruvate, normalizing the metabolite to near baseline levels before slowly rising again. This rapid consumption of pyruvate is not accompanied by a rapid increase in lactate concentration (Fig. S7H), suggesting pyruvate is not simply being reduced to lactate. This pattern appears to be specific to glyoxylate since we previously demonstrated in this model that the effective cyanide-scavenging countermeasure, HCP, does not rapidly normalize pyruvate levels^8^. In contrast, following HCP administration, pyruvate levels continue to rise^8^. To rule out the possibility that glyoxylate is reacting directly with pyruvate to result in its consumption, we analyzed products of glyoxylate and pyruvate in vitro by ESI–MS and could only identify a non-covalent interaction that would be unlikely to contribute to this phenomenon (Supplementary Fig. S6). Interestingly, the glyoxylate-derived metabolite, oxalate, also increases following glyoxylate administration, and its abundance only begins to decrease after the reduction in glyoxylate levels (Fig. 5A). Relative levels of other TCA cycle metabolites are also affected in response to glyoxylate treatment after cyanide exposure (Supplementary Fig. S7). Supplementary Fig. S8–S10 show quantification of pyruvate, glyoxylate, and oxalate levels, respectively, in rabbit serum at baseline. Absolute quantification of pyruvate and glyoxylate in rabbits infused with glyoxylate in the absence of cyanide is also included in Supplementary Fig. S8–S9. Notably, none of these glyoxylate-related metabolites provides similar protection against cyanide toxicity. Specifically, oxalate, whose abundance mirrors that of glyoxylate, provides no protection against cyanide exposure (Fig. 5B), suggesting that while conversion of glyoxylate to oxalate occurs, it is not oxalate per se that provides rescue**.** The unique patterns in pyruvate consumption and oxalate generation suggest that glyoxylate administration induces rapid, profound metabolic changes that are distinct from those induced by known cyanide scavengers.

### Lactate dehydrogenase (LDH) activity is required for glyoxylate-mediated protection against cyanide poisoning

**Figure 6:**
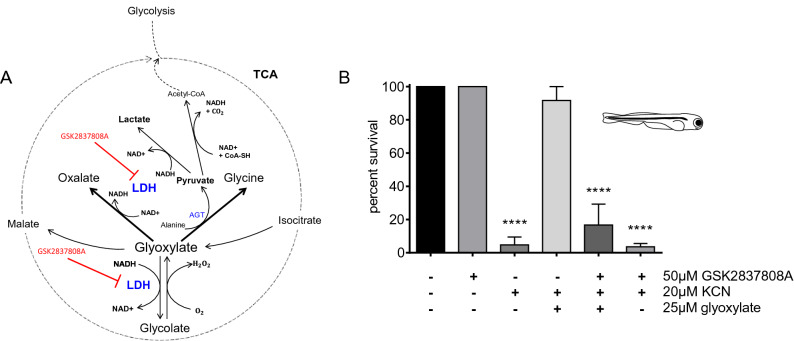
LDH is crucial for glyoxylate metabolism. (**A**) Schematic of glyoxylate and its reactions relative to the TCA cycle. LDH is required for glyoxylate’s oxidation and conversion of pyruvate to lactate. It can also be used for the reduction of glyoxylate. (**B**) Survival of 5 dpf zebrafish 20 h after administration of combinations of the LDH inhibitor GSK2837808A, cyanide, and glyoxylate. Data are presented as mean ± SEM (****p < 0.0001 using one-way ANOVA). There was no significant difference between the untreated control and KCN + glyoxylate and no significant difference between inhibitor + KCN and KCN alone. Each condition had 3 fish per well, N = 3 wells for inhibitor alone, N = 8 for untreated control, N = 7 for KCN alone, N = 4 for KCN + glyoxylate, N = 8 for KCN + glyoxylate + inhibitor, N = 28 for KCN + inhibitor.

Observing that none of the molecules derived from glyoxylate that were tested could provide the same degree of protection as glyoxylate alone, we hypothesized that the glyoxylate mechanism of rescue might be attributable to cofactors involved in the metabolism of glyoxylate. Figure 6A illustrates the link between glyoxylate and the TCA cycle as well as its close relationship to lactate dehydrogenase (LDH) and redox chemistry. Glyoxylate can be oxidized to form oxalate through the reduction of NAD+ to NADH via LDH^21–23^. It can also generate NAD+ through the reduction of pyruvate to lactate along the alanine-glyoxylate amino-transferase (AGT) pathway. Additionally, glyoxylate can be reduced to form glycolate through the oxidation of NADPH to NADP+ by glyoxylate reductase/hydroxypyruvate reductase (GRHPR)^24^, although LDH can also catalyze the reduction of glyoxylate using NADH as a cofactor^21–23^. We used CRISPR-Cas9 to target both isoforms of GRHPR, *grhpra* and *grhprb* in zebrafish. At 5 dpf, we found that glyoxylate’s rescue of cyanide toxicity was unaffected in the first generation mosaic *grhpr* mutant (crispant) fish vs wild type fish (Supplementary Fig. S11, Supplementary Table S3), suggesting that GRHPR does not play a role in glyoxylate’s mechanism of rescue. This turned our attention to the importance of LDH in these reactions and raised the question of whether LDH is required for glyoxylate’s overall cyanide countermeasure capability. To address this question, the survival of zebrafish in the presence of combinations of cyanide, glyoxylate, and the LDH inhibitor GSK2837808A^25^ was tested (Fig. 6B and Supplementary Fig. S12). We found that the inhibitor alone was non-toxic, but that GSK2837808A dramatically attenuated the ability of glyoxylate to rescue cyanide toxicity. We could not identify any reaction products between glyoxylate and GSK2837808A in vitro, suggesting that these results are not due to inactivation of glyoxylate (Supplementary Fig. S13). The results suggest that glyoxylate flux through LDH is critical for mitigating cyanide toxicity.

### Glyoxylate restores cellular redox state

**Figure 7:**
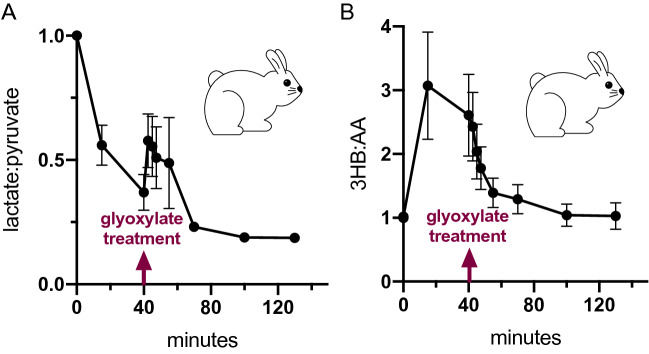
Glyoxylate reverses compartmentalized redox imbalance. (**A**) The ratio of lactate:pyruvate, a surrogate for the ratio of cytosolic NADH to NAD+ and (**B**) the ratio of 3-hydroxybutyrate (3HB) to acetoacetate (AA), a surrogate for the ratio of mitochondrial NADH to NAD+, in serial plasma samples collected from rabbits exposed to a 40 min cyanide infusion followed by i.m. glyoxylate treatment. Data are presented as mean ± SEM, N = 9.

Cyanide poisoning is associated with altered redox states in a compartment-specific manner, with the mitochondrial NADH:NAD+ ratio being elevated relative to normal physiology and the cytosolic NADH:NAD+ ratio being depressed^26,27^. Given the redox reactions catalyzed by LDH using glyoxylate as a substrate and the requisite LDH activity for protection against cyanide toxicity, we hypothesized that LDH and glyoxylate can restore redox balance in both the cytosol and mitochondria through this pathway. To test this hypothesis, baseline and serial blood samples were collected from rabbits in a lethal model of cyanide exposure^28^ followed by i.m. glyoxylate treatment. The ratio of 3-hydroxybutyrate:acetoacetate (3HB:AA) was measured as an established mitochondrial NADH:NAD+ proxy along with lactate:pyruvate ratio as proxy for cytosolic NADH:NAD+ (Fig. 7A,B)^29^. Upon cyanide exposure, rabbit serum exhibited a sharp increase in the surrogate ratio for mitochondrial NADH:NAD+, which was subsequently relieved by glyoxylate treatment, similar to previous observations in zebrafish models of glyoxylate rescue^10^. The cytosolic NADH:NAD+ displayed similar kinetics to that of pyruvate in Fig. 5A, with initial pyruvate accumulation, followed by rapid consumption upon glyoxylate administration, and then gradual accumulation again. These data support the concept that glyoxylate is acting as a metabolic modulator through restoration of cyanide-altered compartment-specific redox states.

### Distinct redox characteristics of glyoxylate confer protective capacity

Glyoxylate-dependent restoration of the redox balance in both the mitochondria and cytosol was further evaluated by comparing these effects with those elicited by molecules with similar redox capacities. To test the relation between redox capacity and protection from cyanide toxicity, we exposed 5 dpf zebrafish to cyanide in the presence of a variety of close structural analogs of glyoxylate (Table 1). Specifically, we focused on comparing the rescue efficacy of metabolites possessing the ability to be oxidized, reduced, form a cyanohydrin, and/or that could be classified as a 2-oxo acid, a preferential LDH substrate^30^. Of the metabolites tested, glyoxylate is the only molecule possessing all of these properties and demonstrated an effective concentration of 1.4E−05 M, ~ 3.6 fold lower than glyceraldehyde (Table 1, Fig. 2A), the next most potent metabolite. Together, these data indicate that glyoxylate’s cyanide toxicity rescue properties are not fully explained by its ability to form a cyanohydrin, but that it may also be acting by normalizing compartment specific redox perturbations. Its efficacy relative to other molecules capable of oxidation, reduction, cyanohydrin formation, or utility as LDH substrates appear to demonstrate that its specific redox characteristics may be central to its ability to protect against cyanide toxicity.

**Table 1:**
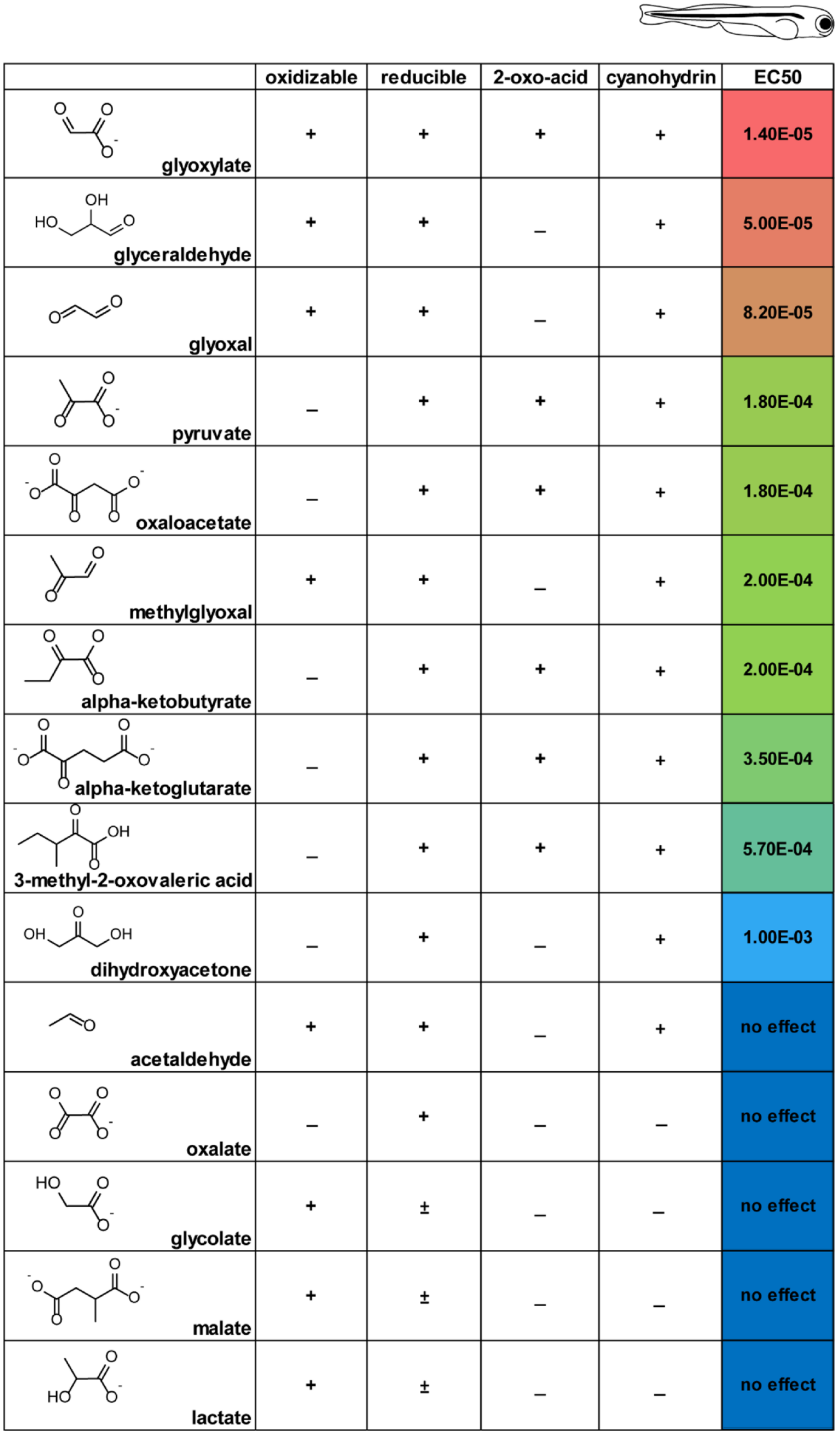
Properties of metabolites with structural similarities to glyoxylate. EC50 values were obtained from data presented in Fig. 5B.

## Discussion

The profound effects of cyanide on primary metabolism are widely known but only partially understood. Cyanide is a known Complex IV inhibitor, causing profound metabolic sequelae for the electron transport chain and energy production. However, the effects on central carbon metabolism have received less than a complete analysis. A broad-based screen of nearly 500 human cofactors and vitamins identified compounds that counteract cyanide toxicity. Glyoxylate became the focus of our studies after we identified this compound as an effective countermeasure against cyanide in a focused zebrafish screen^10^. Our studies here further demonstrate the rescue efficacy in both mouse and rabbit models of cyanide toxicity. Confirmation and follow up studies presented here provide new evidence for the importance of organic metabolites as metabolic rescue agents for cyanide poisoning.

While demonstrating that glyoxylate can react directly with cyanide to form a cyanohydrin, we also observed distinct kinetics and metabolomics in glyoxylate rescue compared with other known cyanide scavengers, suggesting that glyoxylate rescue of cyanide toxicity may not be explained by cyanide scavenging alone. It is possible, however, that these other cyanide scavengers require higher concentrations to achieve a rescue as a result of larger, sterically hindering substitutions at the reactive site^31^. Therefore, glyoxylate may simply be more reactive and form more cyanohydrin at lower concentrations. In plants and fungi, glyoxylate fulfills a key role in a truncated version of the TCA cycle, known as the glyoxylate shunt. In this shunt, isocitrate is lysed to form glyoxylate, which can then be combined with acetyl-CoA to form malate by malate synthase and rejoin the TCA cycle^32,33^. In vertebrates, glyoxylate has clearly been detected, but it is unlikely that the classical glyoxylate shunt occurs^32,34–37^. Nevertheless, glyoxylate can undergo redox reactions through LDH to form oxalate or glycolate, and in the process, produce NADH or NAD+, respectively.

Perhaps the most compelling evidence that glyoxylate rescues cyanide toxicity through a mechanism distinct from known cyanide countermeasures is the rapid change in pyruvate metabolism and redox balance upon treatment with glyoxylate. In the non-lethal rabbit model, an almost immediate conversion of oxygenated hemoglobin to deoxygenated hemoglobin is observed, whereas other known countermeasures, such as alpha-ketoglutarate, normalize this parameter with much slower kinetics. This rapid response to glyoxylate treatment is further demonstrated by the rapid consumption of pyruvate observed in glyoxylate treatment of cyanide-exposed rabbits. Rescue with other cyanide countermeasures, such as HCP, demonstrates no immediate change in pyruvate metabolism after treatment^8^. This immediate consumption of pyruvate, upon treatment with glyoxylate, is not accompanied by a corresponding increase in lactate, a phenomenon that would be expected if pyruvate were being reduced to lactate as occurs during anaerobic metabolism. Rather, the immediate consumption of pyruvate and conversion of oxygenated hemoglobin to deoxygenated hemoglobin is evidence that glyoxylate causes an immediate restoration of electron transport activity.

Cyanide binds to Complex IV of the electron transport chain, preventing electrons from being transferred to the terminal electron receptor, molecular oxygen. This inhibition leads to the inability of the electron transport chain to receive electrons from NADH in the mitochondria and produces an imbalance in the NADH:NAD+ ratio in both the mitochondria and the cytosol. Inhibition of the electron transport chain also leads to “backing up” of the TCA cycle as NADH is not being recycled to NAD+, which is required for multiple steps of the TCA cycle.

We propose that glyoxylate acts as a rapid and effective cyanide countermeasure because of its unique combination of multiple protective mechanisms. First, glyoxylate is capable of directly binding to cyanide. Some of the known cyanide scavenging molecules form cyanohydrins by directly reacting with cyanide through an aldehyde or ketone moiety^38^. The action of glyoxylate scavenging cyanide to form a cyanohydrin could contribute to decreased inhibition of Complex IV of the electron transport chain by preventing cyanide from binding to additional cytochrome c oxidase macromolecules. Furthermore, the rapid restoration of hemoglobin to normal oxygenation levels and immediate consumption of pyruvate observed in our rabbit model upon treatment with glyoxylate suggest that glyoxylate relieves the cyanide block of cytochrome c oxidase and immediately reactivates the electron transport chain.

Second, glyoxylate possesses the capacity to be both oxidized and reduced. As mentioned above, cyanide perturbs the redox balance in the cytosol and mitochondria in opposing directions—glyoxylate can restore the redox balance in both compartments. In our study, we observed that glyoxylate and other molecules with the capacity to be both oxidized and reduced, such as glyceraldehyde, have greater efficacy as cyanide countermeasures than molecules that only can be reduced, such as 2-keto acids (e.g. pyruvate or oxaloacetate). The results here demonstrate that LDH is critical for glyoxylate efficacy, since LDH can reduce glyoxylate to glycolate and generate NAD+ or it can oxidize glyoxylate to oxalate and produce NADH. The direction of the reaction would be dependent on the ambient redox state in the local environment. Therefore, the opposing cyanide-induced redox imbalances in the two compartments could cause LDH to drive glyoxylate redox metabolism in opposing directions in the two compartments (i.e. decreasing NADH:NAD+ ratio in mitochondria and increasing it in cytoplasm). The efficacy of glyoxylate would seem to be dependent on the presence of enzymes capable of oxidizing or reducing glyoxylate in both the cytosol and mitochondria. One possibility is that LDH, whose activity has been reported in both the mitochondria and cytosol^39–42^, is essential for glyoxylate-mediated cyanide rescue. We suggest that this unique combination of glyoxylate and oxidoreductase enzymes, like LDH, that possess the ability to generate both reducing or oxidizing equivalents in the mitochondria and cytosol, provide one mechanism whereby glyoxylate can protect against cyanide toxicity.

One way that restoring redox balance, through the activity of LDH and glyoxylate, might restore the activity of the electron transport chain is through modulating cyanide’s affinity for cytochrome c oxidase. It has been shown that cytochrome c oxidase exists in at least two states with dramatically different rates of reaction with cyanide, including a fully-oxidized state characterized by much slower cyanide binding^43–45^. It is possible that reduction of glyoxylate in mitochondria leads to oxidation of cytochrome c oxidase to the point where it no longer has high affinity for cyanide, preventing further inhibition by cyanide and/or enabling re-initiation of electron transport. However, since cyanide dissociation is generally accepted to be slower than binding^44^, a slow binding rate alone may not fully account for a rapid re-initiation of electron transport activity. Nonetheless, empirical evidence demonstrates that high-affinity cyanide scavengers are associated with immediate clinical improvement, supporting the idea that cyanide dissociation may not be slow in all contexts^46,47^.

Restoring mitochondrial NADH:NAD+ ratio may also modulate the activity of pyruvate dehydrogenase. In a previous study, we demonstrated that inhibition of pyruvate dehydrogenase kinase (PDK) has a protective effect against cyanide toxicity^10^. PDK phosphorylates and inactivates pyruvate dehydrogenase (PDH), the enzyme responsible for converting pyruvate into acetyl Co-A. PDK is localized in the mitochondrial matrix and is activated by an elevated NADH:NAD+ ratio^48^. Glyoxylate and oxidoreductase enzymes, like LDH, have the capacity to act together to normalize the NADH:NAD+ ratio in the mitochondria, thereby reducing the inhibitory activity of PDK on PDH, thus enabling the conversion of pyruvate to acetyl-CoA to fuel the TCA cycle and feed into the electron transport chain. For this increase in electron supply to the electron transport chain to be utilized, some portion of cytochrome c oxidase must be unbound by cyanide. Although it is unlikely that increasing electron supply will drive the flux of electrons through fully inhibited complex IV, many poisoning scenarios involve inhibition of some complex IV units, while others remain uninhibited. Therefore, it is possible that the remaining, uninhibited complex IV units would consume more oxygen in the setting of increased electron supply. Additional investigation will be necessary to uncover if glyoxylate is acting via these proposed models or alternative mechanisms.

The relationship between glyoxylate, LDH, and cyanide toxicity provides a new avenue for future scientific exploration. The discovery of a mechanism whereby toxicity from cyanide poisoning is circumvented by manipulating the redox state of the cytosol and mitochondria expands the armamentarium of potential cyanide countermeasures. Additional oxidoreductases exist in the cytosol and mitochondria that might be targeted to normalize redox imbalance in a subcellular, compartment-specific manner or to bypass discrete functional abnormalities of the electron transport chain^49^. Investigations into identifying substrates that can be used to activate oxidizing pathways in the mitochondria or reducing pathways in the cytosol might lead to the discovery of molecules with similar or improved efficacy compared to glyoxylate, while potentially avoiding some of glyoxylate’s limitations as an intervention. Furthermore, given the evident importance of LDH in glyoxylate’s rescue, exploring other potent LDH substrates in a focused screen could elicit not only further insight into the glyoxylate mechanism of rescue, but also allow for potential discovery of novel drugs for situations where tissue metabolism is compromised.

A potential drawback to the therapeutic use of glyoxylate is the possibility of toxicity in humans. The conversion of glyoxylate to oxalate can lead to the formation of calcium oxalate crystals, as mammals are unable to metabolize oxalate^50,51^. In the current manuscript, no gross abnormalities were observed in glyoxylate treated mice during a 2 week observation period. Nonetheless, we recognize this as an important consideration and one that warrants further exploration, especially in long-term studies and in the setting of compromised renal function.

Existing clinically approved cyanide treatments require i.v. administration of the countermeasure^52^. However, one of the most critical needs for cyanide therapies is the development of a fast-acting, non-intravenous treatment suitable for mass exposure events that is safe and easy to administer. The data presented in this study support the possibility that one dose of i.m. glyoxylate after cyanide exposure can restore metabolic function and prevent cyanide-induced lethality. With no current countermeasure available with similar route of administration, glyoxylate could fill the needed role of such a fast-acting, non-intravenous therapeutic. Additionally, glyoxylate might also be a good candidate for combination therapy, utilizing its metabolic modulation properties and speed of rescue in conjunction with the benefits of a scavenger such as hydroxocobalamin or HCP. The data presented herein, which demonstrate efficacy in three animal models as well as an unexpected role in reactivating mitochondrial metabolism, suggest that glyoxylate should continue to be developed as a countermeasure for cyanide toxicity.

## Methods and materials

### Study design

The investigation presented in this manuscript was initiated to identify small molecules with the capacity to normalize metabolic perturbations that arise as a result of cyanide poisoning. This objective was supported by previous work in which metabolic profiling was used to identify differences in energy metabolism in cyanide-resistant and cyanide-sensitive animals. This previous investigation included a small, directed screen of 48 small molecules (all of which were annotated as modulators of energy metabolism) which identified modulators of the pyruvate dehydrogenase complex, glyoxylate, and others, as potential cyanide countermeasures.

Supported by findings from this previous study, we sought to more comprehensively interrogate cellular metabolic pathways, hypothesizing we would identify candidate molecules in other metabolic processes in the cell, as opposed to solely focusing on energy metabolism. While it was observed previously that glyoxylate protected against cyanide toxicity, the finding that glyoxylate is a uniquely potent cyanide countermeasure amongst ~ 500 human metabolites was quite unexpected.

For this investigation, we utilized high-throughput screening with zebrafish embryos for multiple reasons including: (1) Zebrafish are sensitive to cyanide at the development stage we utilize in this screen (5 dpf). (2) Many physiological features are conserved between zebrafish and humans, and the genes and critical pathways required to develop these features are also highly conserved. (3) A single zebrafish can produce hundreds of isogenic progeny in a single day. (4) Zebrafish embyros can be arrayed in a 96 well format easily for high-throughput screening. Compound library screening was performed with 5 dpf zebrafish embryos, as well as a subset of the validation experiments.

Confirmation studies of the effectiveness of glyoxylate against cyanide toxicity were performed with well-established mouse and rabbit models of cyanide toxicity. Pharmacological studies and mechanistic studies of glyoxylate rescue, each evaluating several variables, were performed using a zebrafish model of cyanide toxicity. Studies characterizing the physiological and metabolic responses to cyanide poisoning and glyoxylate rescue were performed with an established rabbit model of cyanide toxicity. Sample sizes for each experiment varied based on animal availability and are indicated in the body of the manuscript. Specific details of the treatments and measurement techniques used for each experiment are described in the “Materials and methods” section and figure legends. All methods were carried out in accordance with relevant guidelines and regulations, and animal experiments were reported according to ARRIVE guidelines.

### Zebrafish

#### Human metabolite screen

5 dpf TuAB zebrafish embryos were arrayed in 96 well plates with E3 medium (5 mM NaCl, 0.17 mM KCl, 0.33 mM CaCl_2_, 0.33 mM MgCl_2_) and were exposed to 10 µM potassium cyanide (KCN) and either one of 485 human metabolites or DMSO with 3 fish per well. A Beckman Biomek NXP SPAN-8 Automated Liquid Handler was used to aspirate and dispense metabolites that were arrayed in 384-well plates. Fish were screened at 3 h for responsiveness to touch (movement when the plate is tapped) and 24 h for survival as determined by visualization of presence or absence of a heartbeat under a Zeiss SteREO Discovery V8 microscope. This screening process was repeated using the same procedure. Following screening, all zebrafish were euthanized according to IACUC protocol.

#### Seahorse oxygen consumption

For baseline oxygen consumption in Fig. 3, 5 dpf TuAB zebrafish embryos were arrayed in Seahorse XF24 Islet Capture microplates with 300 µL 20 mM HEPES buffered E3 medium with one fish per well. Embryos were treated with 1% DMSO (final concentration) or 100 µM glyoxylate in 1% DMSO (final concentration) and incubated at 28 °C for 15 min. Embryos were then treated with either water or 20 µM KCN. The microplate was then sealed with clear plastic film and incubated at 28 °C for 2 h. After incubation, each embryo was carefully positioned below the islet capture inserts. Oxygen consumption was then measured using Seahorse XF24 Analyzer^53^ at the University of Utah Metabolic Phenotyping Core.

For mitochondrial stress test in Supplementary Fig. S1, 24 hpf TuAB embryos were dechorionated with Pronase at 1 µg/µL at room temperature for 5 min. Embryos were then washed and transferred into fresh 20 mM HEPES buffered E3 pH 7.2. One embryo per well was placed into a 24 well plate with 500 µL of 20 mM HEPES buffered E3 pH 7.2. Embryos were treated with 1% DMSO (final concentration) or 100 µM glyoxylate in 1% DMSO (final concentration) and incubated for 15 min at 28 °C. Embryos were then treated with 100 µM KCN or E3 (vehicle control). The plate was then sealed and incubated for 24 h at 28 °C. At 2 dpf, treated embryos were transferred to Seahorse XF24 Islet Capture microplates, one embryo per well, and positioned below islet capture inserts. Oxygen consumption was then measured using Seahorse XF24 Analyzer at the Brigham and Women’s Hospital Seahorse core.

#### Zebrafish survival with cyanide scavengers or metabolites

5 dpf TuAB zebrafish embryos were arrayed in 96 well plates with 300 µL 20 mM HEPES buffered E3 buffer with 3 fish per well. Embryos were pretreated with metabolites or other compounds of interest dissolved in DMSO or water at the indicated concentrations. Embryos were incubated with these compounds for 15 min at 28 °C. If compounds were solubilized in water, then DMSO was also added to the well so that in every experiment there was a final concentration of 1% DMSO in the well. Embryos were then treated with 20 µM KCN after which the 96 well plates were sealed with clear plastic film and incubated at 28 °C overnight for 20 h. Fish were then assayed for survival by evaluating the presence or absence of a heartbeat. The same procedure was utilized in assessing sulfide and azide toxicity.

For evaluation of lactate dehydrogenase, embryos were pretreated for 10 min with 50 µM GSK2837808A (Cayman, item no. 20626) dissolved in DMSO or DMSO alone such that each well had a final concentration of 1% DMSO, after which, the above procedure was followed with the indicated concentrations of glyoxylate and KCN.

#### Generation of grhpr crispant zebrafish

20 base pair *grhpra* and *grhprb* specific targeting sequences were designed using ChopChop^54^. Oligos were prepared containing the targeting sequences with a 17 base pair SP6 promotor at the 5′ end and a 23 base pair overlap region at the 3′ for a final product of 5′-ATTTAGGTGACACTATA(N_20_)GTTTTAGAGCTAGAAATAGCAAG-3′ (see method of Gagnon et al.^55^). These oligos were annealed and extended via PCR with a constant oligo of 5′-AAAAGCACCGACTCGGTGCCACTTTTTCAAGTTGATAACGGACTAGCCTTATTTTAACTTGCTATTTCTAGCTCTAAAAC-3′ (see method of Shah et al.^56^). Purified PCR products were used as a template in overnight SP6 in vitro transcription reactions at 37 °C using the MEGAscript SP6 transcription kit. Guide RNAs were purified from these reactions via the Zymo RNA Clean & Concentrator kit. Guides were diluted to ~ 400 ng/µL and stored at − 80 °C. 2 µL of each of 5 gRNAs was later combined with 10% by volume Cas9 nuclease and 5% by volume phenol red and incubated at room temperature for 15 min. TuAB zebrafish embryos in the one cell stage were injected with ~ 1.5 nL of the resulting Cas9-gRNA ribonucleoprotein.

### Mouse

#### Mouse model of cyanide toxicity

Mice are small enough that they can be exposed to cyanide gas within a sealed chamber. The mice were exposed to 587 ppm HCN gas for 15 min, injected with test antidote in the gastrocnemius muscle, and then re-exposed to the gas for 25 min. This model assumes about 15 min are required for emergency medical personnel to arrive at a disaster scene, and another 25 min are required to treat and evacuate the victims. The mice are observed continuously during the cyanide exposure period and for 60 min after cyanide exposure, paying special attention to their breathing. Any animal that is apneic for more than 1 min is considered to have expired. Surviving animals were returned to their cages and observed daily for two weeks, at which time they were euthanized by breathing 30% carbon dioxide for 2–3 min, followed by exsanguination via an intracardiac puncture. As required by IACUC, the mice were anesthetized by injecting isoflurane into the chamber to a final concentration of 2%; at 30 °C, the isoflurane rapidly vaporizes and anesthetizes the mice.

### Rabbit

#### General preparation

Twenty pathogen-free New Zealand White rabbits weighing 3.5–4.5 kg (Western Oregon Rabbit Supply) were used. All procedures were reviewed and approved by the University of California, Irvine, Institutional Animal Care and Use Committee (IACUC). The methods of diffuse optical spectroscopy (DOS) monitoring have been described previously and are summarized here^57^.

#### Animal preparation

Animals were anesthetized with an i.m. injection of a 2:1 ratio of ketamine HCl (100 mg/mL, Ketaject, Phoenix Pharmaceutical Inc., St. Joseph, MI): Xylazine (20 mg/mL, Anased, Lloyd Laboratories, Shenandoah, IA) at a dose of 0.75 cc/kg, using a 23 gauge 5/8 in. needle. After the i.m. injection, a 23 gauge 1 in. catheter was placed in the animals’ marginal ear vein to administer continuous i.v. anesthesia. The animals were intubated with a 3.0 cuffed endotracheal tube secured by a gauze tie and were mechanically ventilated (dual phase control respirator, model 32A4BEPM-5R, Harvard Apparatus, Chicago, IL) at a respiratory rate of 20 to 22 breaths/min, a tidal volume of 60 cc, and fraction of inspired oxygen (FiO_2_) of 100%. A pulse oximeter (Biox 3700 Pulse Oximeter, Ohmeda, Boulder, CO) with a probe placed on the tongue was used to measure arterial blood oxygen saturation (SpO_2_) and heart rate. Blunt dissection was performed to isolate the femoral artery and vein on the left thigh for blood sampling, cyanide infusion, and systemic pressure monitoring. The antidote i.m. injection site was prepared by exposing the right inner arm muscle.

#### Lethal level cyanide poisoning

NaCN solution was prepared by dissolving 20 mg NaCN (SigmaAldrich, St. Louis, MO) in 60 mL of 0.9% saline in a 60 cc plastic syringe and was given i.v. by pump at the rate of 1 mL (0.33 mg) per min. After 30 min of cyanide infusion at 100% oxygen to pre-load cyanide, oxygen supply was switched to room air and the respiratory rate on the ventilator was reduced down to 18–20 breaths/min. This results in a lethal condition as cyanide infusion continues and blood pressure falls. Antidote or saline was delivered when the trigger target blood pressure of 40–58 mmHg systolic was reached. The cyanide infusion was discontinued 30 min after administration of the treatment. This protocol resulted in rabbits receiving 22–26 mg of total cyanide, which, in this model, is lethal in 80% of untreated rabbits. Animals are considered non-survivors and euthanized if systolic blood pressure drops below 20 mmHg at any time during the experiment. On completion of the experiment (90 min), the surviving animals were euthanized with an i.v. injection of 1.0 cc Euthasol (390 mg pentobarbital sodium, 50 mg phenytoin sodium; Vibrac AH, Inc, Fort Worth, TX) administered through the marginal ear vein.

#### Glyoxylate delivery

Glyoxylate (200 mg) in 2 mL of 0.9% NaCl (N = 9) or 1 cc 0.9% NaCl (N = 11) was delivered i.m. at the exposed upper arm muscle injection site at the blood pressure trigger point as described. The glyoxylate dose was divided into 2 × 1 cc syringes and delivered at adjacent muscle sites to avoid excessive single site volume.

#### Data collection

Arterial and venous blood gasses and venous blood cyanide levels were measured at baseline, 15 and 25 min of cyanide infusion. Additional blood samples were collected after glyoxylate injection, including 2.5, 5, 7.5, 10, 15, 30, 45, 60 and 90 min post-injection samples. During this time, DOS measurements were taken continuously.

#### Non-invasive measurements using diffuse optical spectroscopy (DOS)

DOS measurements were obtained through a fiber optic probe with a light diode emitter and detector at a fixed distance (10 mm) from the source fiber, which was placed on the shaved surface of the right inner thigh of the animal. The broadband DOS system we constructed^15^ combines multi-frequency domain photon migration with time-independent near infrared spectroscopy to accurately measure bulk tissue absorption and scattering spectra. It employs six laser diodes at discrete wavelengths (661, 681, 783, 805, 823, and 850 nm), and a fiber coupled avalanche photo diode (APD) detector (Hamamatsu high-speed APD module C5658, Bridgewater, NJ) for the frequency domain measurements. The APD detects the intensity-modulated diffuse reflectance signal at modulation frequencies between 50 to 550 MHz after propagation through the tissue. Absorption and reduced scattering coefficients were measured directly at each of the six laser diode wavelengths using frequency-dependent phase and amplitude data. Reduced scattering coefficients were calculated as a function of wavelength throughout the NIR region by fitting a power-law to six reduced scattering coefficients. Steady-state acquisition was accomplished using a broadband reflectance measurement from 650 to 1000 nm that follows frequency domain measurements using a tungsten-halogen light source (Ocean Optics HL-2000, Dunedin, Fl) and a spectrometer (BWTEK BTC611E, Newark, DE). Intensity of the steady-state reflectance measurements were calibrated to the frequency domain values of absorption and scattering to establish the absolute reflectance intensity^57–59^. Tissue concentrations of oxy- and deoxyhemoglobin and cytochrome c oxidase redox state (ratio of oxidized to reduced cytochrome c oxidase) were calculated by a linear least squares fit of the wavelength-dependent extinction coefficient spectra of each chromophore as previously described^14,15,57,60^. We used oxy- and deoxyhemoglobin absorption spectra reported by Zijlstra et al.^61^ for subsequent fitting and analysis.

### Mass spectrometry

#### Chromatography and mass spectrometry methods

Metabolites were extracted using acetonitrile/methanol (75:25; v/v) containing deuterated internal standards (25 μM thymine-d_4_ [Sigma-Aldrich], 10 μM inosine-^15^N_4_ [Cambridge Isotope Laboratories], 10 μM citrulline-d_7_ [Sigma-Aldrich], 25 μM phenylalanine-d_8_ [Cambridge Isotope Laboratories], and 10 μM valine-d_8_ [Sigma-Aldrich]). The samples were separated using a 2.1 × 100 mm 3.5-μm Xbridge amide column (Waters). Mobile phase A was 95:5 (v/v) water/acetonitrile, with 20 mM ammonium acetate and 20 mM ammonium hydroxide (pH 9.5). Mobile phase B was acetonitrile. For amide-negative mode, the chromatography system consisted of a 1260 Infinity autosampler (Agilent) connected to a 1290 Infinity HLPC binary pump system (Agilent). The eluents were detected in negative mode on a coupled 6490 QQQ mass spectrometry equipped with an electrospray ionization source. The settings were as follows: sheath gas temperature, 400 °C; sheath gas flow, 12 L/min; drying gas temperature, 290 °C; drying gas flow, 15 L/min; capillary, 4000 V; nozzle pressure, 30 psi; nozzle voltage, 500 V; and delta EMV, 200 V. Detailed methods have been described previously^62^.

#### Isotope distribution

Reaction products were generated by preparing unlabeled (12C14N) and isotope-labeled (13C15N) sodium cyanide solutions at 0.135 M, pH = 7.4. Next a stock solution of 20 mM glyoxylate was prepared in water. Glyoxylate was added to the cyanide solutions generating a final concentration of 20 µM glyoxylate and incubated (1 µL of 20 mM glyoxylate, pH = 7.4, was added to 1 mL of cyanide, pH = 7.4) for 2 h. The resulting mixture was diluted tenfold with HPLC-grade methanol and then infused directly into an Agilent 6550 iFunnel Q-TOF Mass Spectrometer equipped with a dual AJS-ESI source. The MS-only full scan and/or targeted MS/MS scan in positive mode was acquired through Agilent MassHunter Workstation LC/MS Data Acquisition software (version B.05.01). MS1 and MS2 spectra were obtained and compared using Agilent MassHunter Workstation Qualitative Analysis software (version B.06.00) for peak identification.

#### Statistics

The log-rank test (Mantel–Cox) statistical method was used to compare the survival distributions of glyoxylate-treated versus saline-treated rabbits in the lethal cyanide model. The log-rank test (Mantel–Cox) was also used to compare survival between glyoxylate and saline-treated mice.

Fish survival data are presented as mean ± SEM. Zebrafish LDH inhibition and oxygen consumption p values were obtained using one-way ANOVA. Best fit curves were obtained using Graphpad Prism8 with a sigmoidal, 4PL non-linear fit where X is concentration. In all zebrafish studies, the subject number, N, refers to the number of wells for a given condition. Each well contained 3 zebrafish.

#### Study approval

Zebrafish studies were reviewed and approved by the University of Utah Institutional Animal Care and Use Committee. Rabbit studies were reviewed and approved by the University of California, Irvine, Institutional Animal Care and Use Committee. Mouse studies were approved by the UCSD Institutional Animal Care and Use Committee.

## Supplementary Information

Supplementary Information.

## Data Availability

All data associated with this study are available in the main text or the supplementary materials.
