# Supplementary material for: Glyoxylate protects against cyanide toxicity through metabolic modulation
Source: Sci Rep. 2022 Mar 23;12:4982. doi: 10.1038/s41598-022-08803-y (PMC8943054; doi:10.1038/s41598-022-08803-y)
Supplement: Supplementary file 1 — Supplementary Information. [file 41598_2022_8803_MOESM1_ESM.pdf]

SUPPLEMENTARY MATERIAL

TITLE: Glyoxylate protects against cyanide toxicity through metabolic modulation

ONE SENTENCE SUMMARY: Glyoxylate, a human metabolite, corrects cyanide-induced metabolic perturbations, rapidly protecting against cyanide toxicity in three animal models.

AUTHORS:
Jason R. Nielson[1], Anjali K. Nath[2], Kim P. Doane[1], Xu Shi[2], Jangwoen Lee[3], Emily G. Tippetts[1], Kusumika Saha[4], Jordan Morningstar[2], Kevin G. Hicks[5], Adriano Chan[6], Yanbin Zhao[4], Amy Kelly[4], Tara B. Hendry-Hofer[7], Alyssa Witeof[7], Patrick Y. Sips[8], Sari Mahon[3], Vikhyat S. Bebarta[7], Vincent Jo Davisson[9], Gerry R. Boss[6], Jared Rutter[5], Calum A. MacRae[4], Matthew Brenner[3], Robert E. Gerszten[2], Randall T. Peterson[1]*

AFFILIATIONS:
[1] Department of Pharmacology and Toxicology, College of Pharmacy, University of Utah, Salt Lake City, UT 84112 USA.

[2] Department of Cardiology, Beth Israel Deaconess Medical Center, Boston, MA 02115, USA. Broad Institute, Cambridge, MA 02142, USA.

[3] Beckman Laser Institute and Department of Medicine, University of California, Irvine, CA 92697, USA.

[4] Division of Cardiovascular Medicine, Brigham and Women's Hospital, Boston, MA 02115, USA.

[5] Department of Biochemistry and Howard Hughes Medical Institute, University of Utah, Salt Lake City, USA.

[6] Department of Medicine, University of California, San Diego, CA 92093, USA.

[7] Department of Emergency Medicine, University of Colorado School of Medicine, Aurora, CO 80045, USA.

[8] Department of Biomolecular Medicine, Ghent University, 9000 Ghent, Belgium.

[9] Department of Medicinal Chemistry and Molecular Pharmacology, Purdue University, West Lafayette, IN 47907, USA.

*To whom correspondence should be addressed: Randall T. Peterson, randall.peterson@pharm.utah.edu

Table S1. Compounds tested in metabolite library.

Common Compound Name				
((2R,3S,5R)-3-hydroxy-5-(6-hydroxy-9H-purin-9-yl) tetrahydrofuran-2-yl)methyl phosphate	5,6-Dihydroxyindole	D-Arabinonic acid	L-(−)-Arabitol	Phospho(enol)pyruvic acid
	5-aminoimidazole-4-carboxamide-1-β-Dribofuranosyl 5'-monophosphate	D-Aspartic acid	L-(−)-Fucose	Phosphocholine
(−)-Epinephrine		Decanoic acid	L-(−)-Malic acid	Phosphocreatine
(−)-Riboflavin	5-Hydroxy-L-tryptophan	Delta-Hexalactone	L-(+)-Arabinose	Phytic acid
(±) - 3- Methyl- 2- oxovaleric acid	5-Methoxy-3-indoleacetic acid	D-Erythrose 4-phosphate	L-(+)-Lactic acid	PIP2 06:0
(±)-Carnitine	5-Methoxytryptamine	D-erythro-Sphingosine C-15	L-2-Aminobutyric acid	PIP3 06:0
(2R)-Glycerol-O-β-D-galactopyranoside	5-Methyltetrahydrofolic acid	D-Fructose 1,6-bisphosphate	L-Alanine	Porphobilinogen
(4R)-4-Hydroxy-L-glutamic acid	5-Methyluridine	D-Fructose 6-phosphate	L-allo-Isoleucine	Progesterone
(6S)-Tetrahydrofolic Acid	5-Phospho-D-ribose 1-diphosphate	DG 08:0	L-Anserine	Propiolic acid
(9cis)-retinoic acid	6-(Dimethylamino)purine	D-Gluconic acid	L-Arginine	Propionic acid
(hexanoylamino)acetic acid	6-deoxy-L-galactonic acid	D-Glucosamine 6-phosphate	L-Ascorbic acid	Propylene glycol
(isobutyrylamino)acetic acid	6-Phosphogluconic acid	D-Glucose 6-phosphate	L-Asparagine	Prostaglandin D2
(L)-Dehydroascorbic acid	7,8-Dihydroneopterin	D-Glucuronic acid	L-Aspartic acid	Prostaglandin E2
(propionylamino)acetic acid	9-cis-Retinal	D-Glutamic acid	L-Carnosine	Prostaglandin I2
(R)-(+)-2-Pyrrolidone-5-carboxylic acid	Acetaldehyde	D-Glutamine	L-Citrulline	Protoporphyrin IX
(R)-2,3-dihydroxypropanoic acid	Acetic acid	D-Glyceraldehyde 3-phosphate solution	L-Cystathionine	PS(6:0/6:0)
(R)-2-hydroxy-4-methylpentanoic acid	Acetoacetyl coenzyme A	D-Glycerol 1-phosphate	L-Cysteic acid	Putrescine
(R)-3-Hydroxybutyric acid	Acetyl coenzyme A	Dihydrofolic acid	L-Cysteine	PVSQ-O-GlcNAC
(R)-beta-hydroxyisobutyrate	Acetylamino-acetic acid	Dihydrouracil	L-Cystine	Pyridoxal
(R)-Pantetheine	Acetylcholine chloride	Dihydroxyacetone	L-Dihydroorotic acid	Pyridoxal 5′-phosphate hydrate
(S)-3-Hydroxybutyric acid	Adenine	Dihydroxyacetone phosphate	L-Glutamic acid	Pyridoxamine
(S)-beta-hydroxyisobutyrate	Adenosine	DL-2-Amino-3-phosphonopropionic acid	L-Glutamine	Pyridoxamine-5'-phosphate (Vitamin B6)
[(2-methylbutanoyl)amino]acetic acid	Adenosine 3',5'-cyclic monophosphate	DL-2-Aminoadipic acid	L-Glutathione oxidized	Pyridoxine
{[(4-hydroxyphenyl)acetyl]amino}acetic acid	Adenosine 3',5'-diphosphate	DL-3,4-Dihydroxymandelic acid	L-Glutathione reduced	Riboflavin 5′-monophosphate
1,3-Diaminopropane	Adenosine 3'-phosphate 5'-phosphosulfate	DL-3,4-Dihydroxyphenyl glycol	L-Gulonic acid γ-lactone	S-(5'-Adenosyl)-L-homocysteine
1A,7A-dihydro-1A-methyl-7A-[(2E,6E,10E)-3,7,11,15tetramethyl-2,6,10,14-hexadecatetraen-1-YL] napth[2,3-B]oxirene-2,7-dione	Adenosine 5′-diphosphate	DL-3-Aminoisobutyric acid	L-Histidine	S-(5'-Adenosyl)-L-methionine
	Adenosine 5′-monophosphate	DL-3-Hydroxy-3-methylglutaryl coenzyme A	L-Homocitrulline	Sarcosine
	Adenosine 5′-triphosphate	DL-3-Ureidoisobutyric acid	L-Isoleucine	Serotonin
1-methyl-4-oxo-1,4-dihydropyridine-3-carboxamide	Adenosine 5'-phosphosulfate	DL-4-Hydroxy-3-methoxymandelic acid	Lithium acetoacetate	S-Lactoylglutathione
1-Methylhistamine	Adenylosuccinic acid	DL-5-Hydroxylysine	Lithium potassium acetyl phosphate	Sodium 3-methyl-2-oxobutyrate
1-Methyl-L-histidine	Adipic acid	D-Lactaldehyde solution	Lithium β-hydroxypyruvate	Sodium glycochenodeoxycholate
1-Methylnicotinamide	Adonitol	D-Lactose	L-Kynurenine	Sodium mercaptopyruvate
2-(hydroxymethyl)butanoic acid	Agmatine	DL-beta-Hydroxybutyryl coenzyme A	L-Leucine	Sodium phenylpyruvate
2-(phosphonooxy) acetic acid	all trans-Retinal	DL-Homocysteine	L-Lysine	Sodium pyruvate
2,3-Diphospho-D-glyceric acid	Aminoacetone	DL-Homocystine	L-Methionine	Sodium taurochenodeoxycholate
2,3-Pyridinedicarboxylic acid	Ammonium carbamate	DL-Isocitric acid	L-Ornithine	Spermidine
2,5-Dihydroxybenzaldehyde	AMP-PNP	DL-Mandelic acid	L-Phenylalanine	Spermine
2,5-Dihydroxybenzoic acid	Anthranilic acid	DL-Norepinephrine	L-Pipecolic acid	Sphingosine 1-phosphate
2-[(2-Phenylacetyl)amino]acetic acid	Arachidonic acid	D-Mannose 6-phosphate	L-Proline	Stachyose
2′-Deoxyadenosine	Asp-Phe	D-Mannose-6-phosphate	L-Pyroglutamic acid	Succinic acid
2′-Deoxyadenosine 5′-diphosphate	Benzoic acid	D-myo-Inositol-1,4,5-triphosphate	L-Saccharopine	Succinyl coenzyme A
2′-Deoxyadenosine 5′-monophosphate	Betaine	Dodecanedioic acid	L-Serine	Sucrose
2′-Deoxyadenosine 5′-triphosphate	Betaine aldehyde	Dopamine hydrochloride	L-Threonine	Taurine
2′-Deoxycytidine	Bilirubin	D-Ornithine	L-Thyroxine	Taurocholate
2′-Deoxycytidine 5′-monophosphate	Biliverdin	D-Pantothenic acid	L-Tryptophan	Thiamine hydrochloride
2′-Deoxyguanosine	Biocytin	D-Proline	L-Tyrosine	Thiamine monophosphate
2′-Deoxyguanosine 5′-monophosphate	Biotin	D-Ribose 1-phosphate	L-Valine	Thiamine pyrophosphate
2′-Deoxyinosine	bis[(aminocarbonyl)amino]acetic acid	D-Ribose 5-phosphate	Lyso-Sphingomyelin	Thymidine
2′-Deoxyuridine	Butyric acid	D-Ribulose 5-phosphate	L-α-Glycerophosphorylcholine	Thymidine 5′-monophosphate
25-Hydroxycholesterol	Butyryl coenzyme A	D-Saccharic acid	L-α-Hydroxyglutaric acid	Thymidine 5'-triphosphate
2-Amino-3-methoxybenzoic acid	C-2 Ceramide	D-Sedoheptulose 7-phosphate	Maleic acid	Thymine
2'-Deoxycytidine 5'-diphosphate	C2 Ceramide (D18:1/2:0)	D-Serine	Malonyl coenzyme A	trans-4-Hydroxy-L-proline
2'-Deoxycytidine 5'-triphosphate	C2 Dihydroceramide	D-Sorbitol	Melatonin	trans-beta-Hydromuconic acid
2-Deoxy-D-ribonic acid	Cadaverine	D-Tagatose 6-phosphate	Melibiose	Tryptamine
2-Deoxy-D-ribose	Carbamoylphosphate	Dulcitol	Methanol	Tyramine
2'-Deoxyguanosine 5'-diphosphate	CDP-ethanolamine	dUTP	Methylglyoxal solution	Uracil
2'-Deoxyguanosine 5'-triphosphate	CDP-GLYCEROL	D-Xylulose	Methylmalonic acid	Urea
2-Deoxyribose 5-phosphate	chenodeoxycholate	D-Xylulose-5-phosphate	Mucic acid	Uric acid
2'-Deoxyuridine 5'-monophosphate	Cholic acid	D-α-Hydroxyglutaric acid	myo-Inositol	Uridine
2-Deoxy-α-D-ribose 1-phosphate	Choline	Ethanolamine	N-(2-furoyl)glycine	Uridine 5′-monophosphate
2-Hydroxy-3-methylbutyric acid	Choline glycerophosphate	Ethylene glycol	N,N'-Diacetylchitobiose	Uridine 5'-diphosphate
2-hydroxy-3-methylpentanoic acid	Cinnabarinic Acid	Ethylmalonic acid	N,N-Dimethylglycine	Uridine 5'-diphosphogalactose
2-Hydroxybutanoic acid	cis-4-Hydroxy-D-proline	Flavin adenine dinucleotide	N-Acetyl-5-hydroxytryptamine	Uridine 5'-diphosphoglucose
2-Hydroxyisocaproic acid	cis-Aconitic acid	Folic acid	N-Acetyl-Asp-Glu	Uridine 5'-diphosphoglucuronic acid
2-Hydroxyphenylacetic acid	Citramalate	Folinic acid	N-Acetyl-D-galactosamine	Uridine 5'-diphospho-N-acetylgalactosamine
2-Ketobutyric acid	Citric acid	Fumaric acid	N-Acetyl-D-glucosamine	Uridine 5'-diphospho-N-acetylglucosamine
2-Methylglutaric acid	Coenzyme A	Galactinol	N-Acetyl-D-glucosamine 6-phosphate	Uridine 5'-triphosphate
2-Oxoadipic acid	Coenzyme B12	Glutaconic acid	N-Acetyl-D-mannosamine	Vitamin K1 2,3-epoxide
3,3′,5-Triiodo-L-thyronine	Coenzyme Q1	Glutaric acid	N-Acetyl-L-alanine	Xanthine
3,3-Dimethylacrylic acid	Coenzyme Q10	Glycerol	N-Acetyl-L-aspartic acid	Xanthosine dihydrate
3,4-Dihydroxy-L-phenylalanine	Cortisone	Glycine	N-Acetyl-L-glutamic acid	Xanthurenic acid
3,4-Dihydroxyphenylacetic acid	Creatine	Glycocholate	N-Acetylmuramic acid	XMP
3,5-Diiodo-L-thyronine	Crotonoyl coenzyme A	Glycolic acid	N-Acetylneuraminic acid	Xylitol
3,5-Diiodo-L-tyrosine dihydrate	Cys-Gly	Glyoxylate	N-Acetylputrescine	α-D-Galactose 1-phosphate
3-Aminopiperidin-2-one	Cysteamine	Guanidineacetic acid	N-Acetyl-α-D-glucosamine 1-phosphate	α-D-Glucose 1,6-bisphosphate
3'-Dephosphocoenzyme A	Cytidine	Guanine	Nepsilon-Acetyl-L-lysine	α-D-Glucose 1-phosphate
3-Furoic acid	Cytidine 5′-monophosphate	Guanosine	N-Formylanthranilic acid	α-D-glucose-1-phosphate
3-Hydroxy-3-methylglutaric acid	Cytidine 5'-diphosphate	Guanosine 3',5'-cyclic monophosphate	N-Glycolylneuraminic acid	α-Ketoglutaric acid
3-Hydroxyanthranilic acid	Cytidine 5'-diphosphocholine	Guanosine 5′-monophosphate	Nicotinamide	α-Keto-γ-(methylthio)butyric acid
3-Hydroxy-DL-kynurenine	Cytidine 5'-triphosphate	Guanosine 5'-diphosphate	Nicotinamide hypoxanthine dinucleotide	β-Alanine
3-hydroxypentanedioic acid	Cytidine-5'-monophospho-N-acetylneuraminic acid	Guanosine 5'-diphospho-D-mannose	Nicotinic acid	β-Estradiol
3-Hydroxypropionic acid, 30%solution in water	D- Erythrono- 1,4- lactone	Guanosine 5'-triphosphate	N-Isovalerylglycine	β-L-Fucose 1-phosphate
3-Iodo-L-tyrosine	D-(−)-3-Phosphoglyceric acid	Hippuric acid	N-Propionyl coenzyme A	β-Nicotinamide adenine dinucleotide
3-Methoxytyramine	D-(−)-Fructose	Histamine dihydrochloride	N-α-Acetyl-L-lysine	β-Nicotinamide adenine dinucleotide 2'-phosphate reduced
3-Methylglutaconic acid, mixture of E and Z isomers	D-(−)-Lactic acid	Homogentisic acid	Nα-Acetyl-L-ornithine	β-Nicotinamide adenine dinucleotide phosphate
3-Methylglutaric acid	D-(−)-Ribose	Homovanillic acid	O-Acetyl-L-carnitine	β-Nicotinamide mononucleotide
3-Sulfopyruvic acid	D-(+) Glucose	Hydroquinone	Octanoic acid	γ-Aminobutyric acid
3-Ureidopropionic acid	D-(+)-Arabitol	Hydroxypyruvic acid phosphate	Octanoyl coenzyme A	γ-Glu-Cys
4,6-Dioxoheptanoic acid	D-(+)-Cellobiose	Hypotaurine	O-Phospho-L-serine	
4-Guanidinobutyric acid	D-(+)-Galactose	Hypoxanthine	O-Phosphorylethanolamine	
4-hydroxy-2-oxoglutaric acid	D-(+)-Gluconic acid δ-lactone	Indole-3-acetic acid	Orotic acid	
4-Hydroxybenzoic acid	D-(+)-Glucosamine	Indole-3-pyruvic acid	Orotidine 5'-monophosphate	
4-Hydroxyphenylacetic acid	D-(+)-Glucuronic acid γ-lactone	Inosine	Oxaloacetic acid	
4-Hydroxyphenylpyruvic acid	D-(+)-Glyceraldehyde	Inosine 5′-monophosphate	P1,P3-Di(adenosine-5') triphosphate	
4-Imidazoleacetic acid	D-(+)-Maltose	Inosine 5'-diphosphate	PA(6:0/6:0)	
4-Imidazoleacrylic acid	D-(+)-Mannose	Inosine 5'-triphosphate	p-Benzoquinone	
4-Methyl-2-oxovaleric acid	D-(+)-Raffinose	Isomaltose	PC(3:0/3:0)	
4-Phenylbutyric acid	D-(+)-Trehalose	Isovaleric acid	PE(6:0/6:0)	
4-Pyridoxic acid	D-(+)-Xylose	Isovaleryl coenzyme A	PG(6:0/6:0)	
5,6-Dihydro-5-methyluracil	D,L-Metanephrine	L-(-)-Arabitol	Phenethylamine	

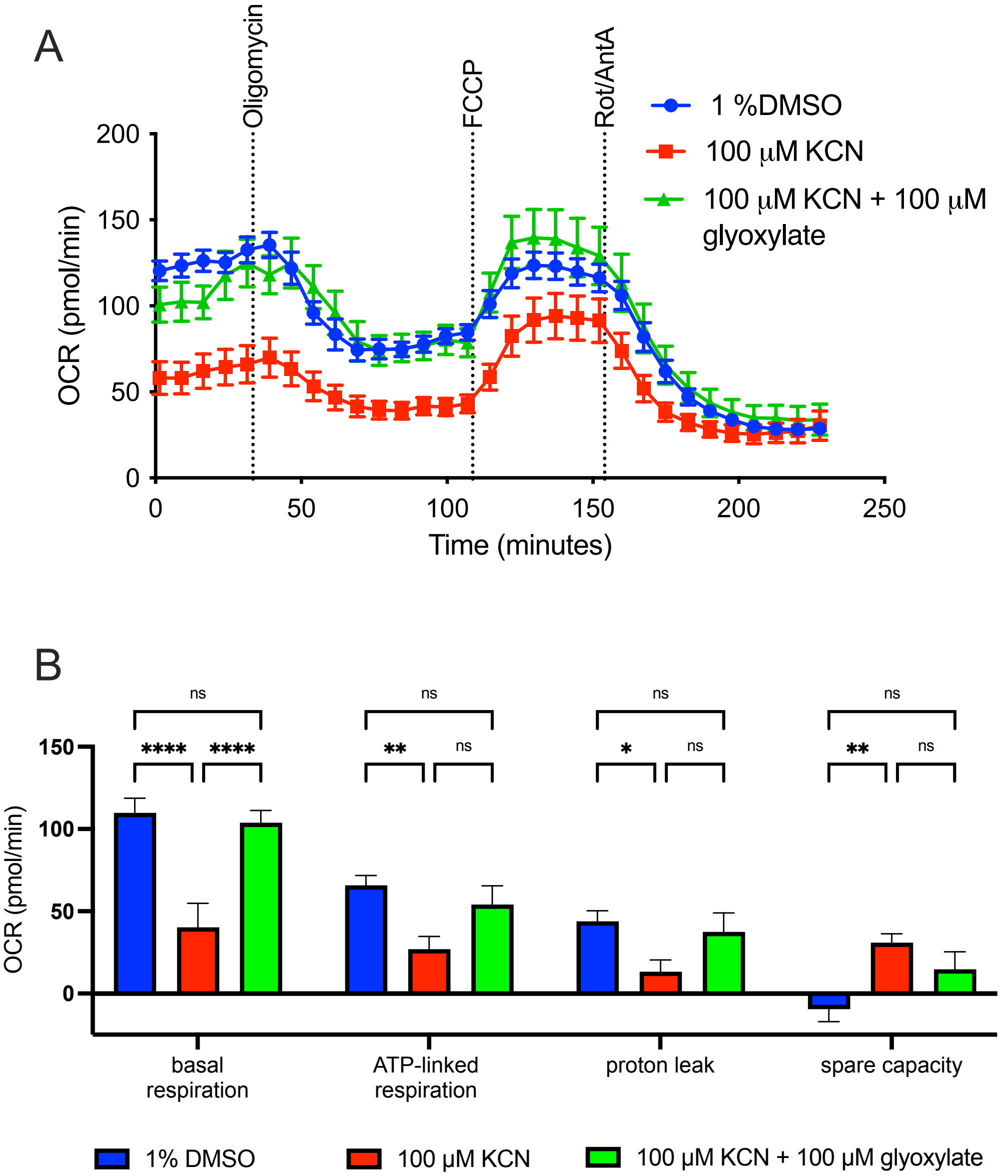

Fig. S1. Mitochondrial stress test in zebrafish model of cyanide toxicity with glyoxylate treatment. A) Oxygen consumption rates of 2 dpf zebrafish embryos arrayed in Seahorse XF24 Islet Capture microplates (1 fish/well) were measured with a Seahorse XF24 Analyzer after treatment with either DMSO, 100 μM KCN or 100 μM KCN + 100 μM glyoxylate. 12 μM oligomycin was added to each well at 33.39 minutes (after the 5th reading), 2.5 μM FCCP was added to each well at 108.74 minutes (after the 15th reading), and 1 μM rotenone and antimycin A were added at 153.99 minutes (after the 21st reading). n=13 for 1% DMSO treated fish. n=10 for 100 μM KCN treated fish. n=8 for 100 μM KCN + 100 μM glyoxylate treated fish. Mean ± SEM shown. **B)** Basal respiration, ATP-linked respiration, proton leak, and spare capacity calculated from raw data in A. * $p < 0.05$, ** $p < 0.01$, **** $p < 0.0001$ using two-way ANOVA.

Table S2. Mass spectral analysis of species produced by the reaction between sodium cyanide and glyoxylate

MS1	MS1	Composition	Isotope shift
72.9939	72.9940	$C_2O_3H_2^-$	0
100.0043	102.0047	$C_3O_3NH_2^-$	2
168.9764	168.9757	$C_4O_6H_4Na^-$	0
195.9865	197.9872	$C_5H_4NO_6Na^-$	2
222.9974	226.9988	$C_6H_5N_2O_6Na^-$	4

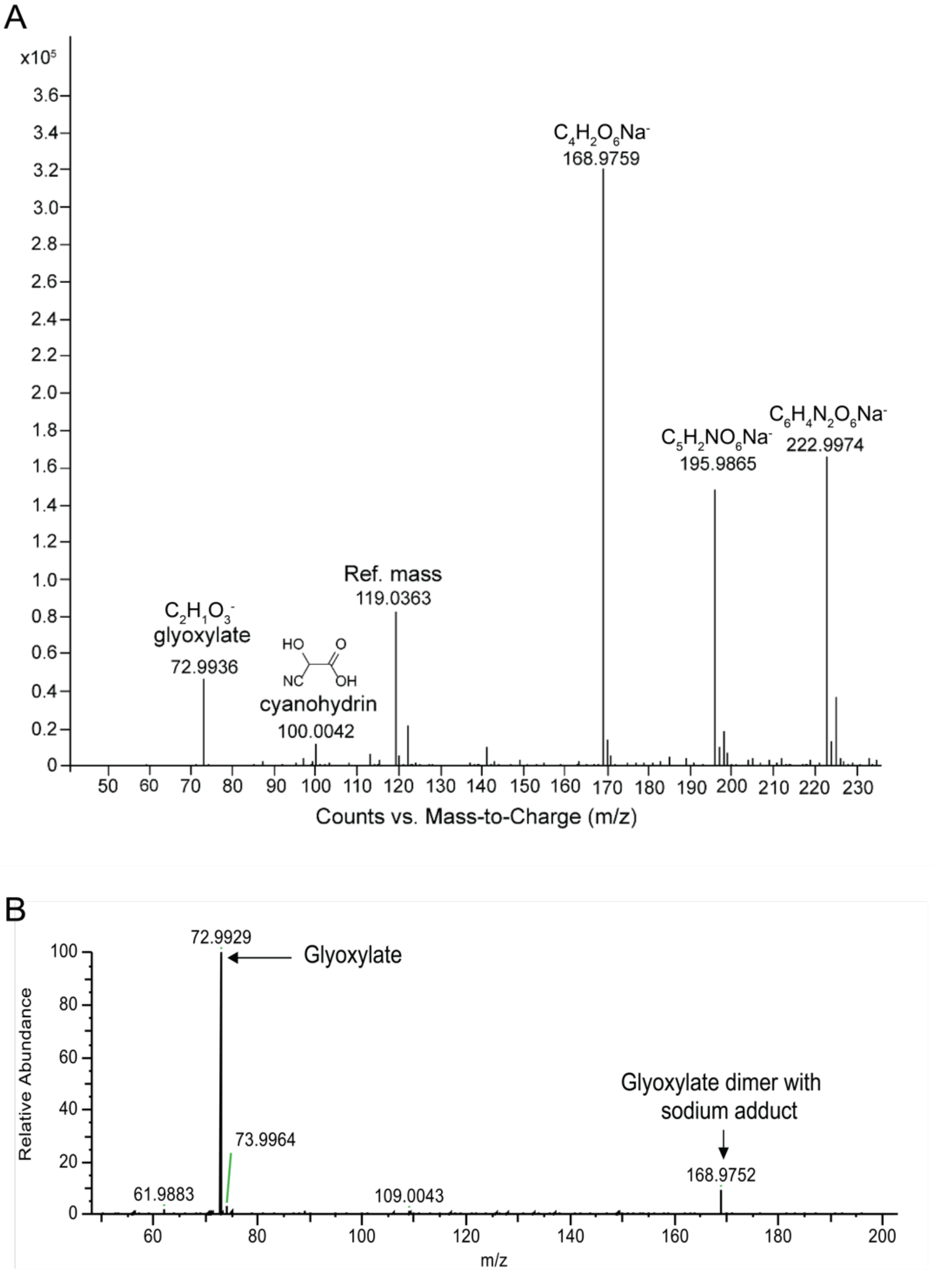

Fig. S2. Identification of the products generated by the reaction of glyoxylate and cyanide. **A)** To decipher the chemical species produced when glyoxylate reacts with cyanide, we used ESI-MS. The most abundant ion signals detected were at m/z = 195 and 222 corresponding to the glyoxylate bound to 1 or 2 cyanide anions, respectively. **B)** Glyoxylate, in the absence of cyanide, can form a dimer (m/z = 168.9).

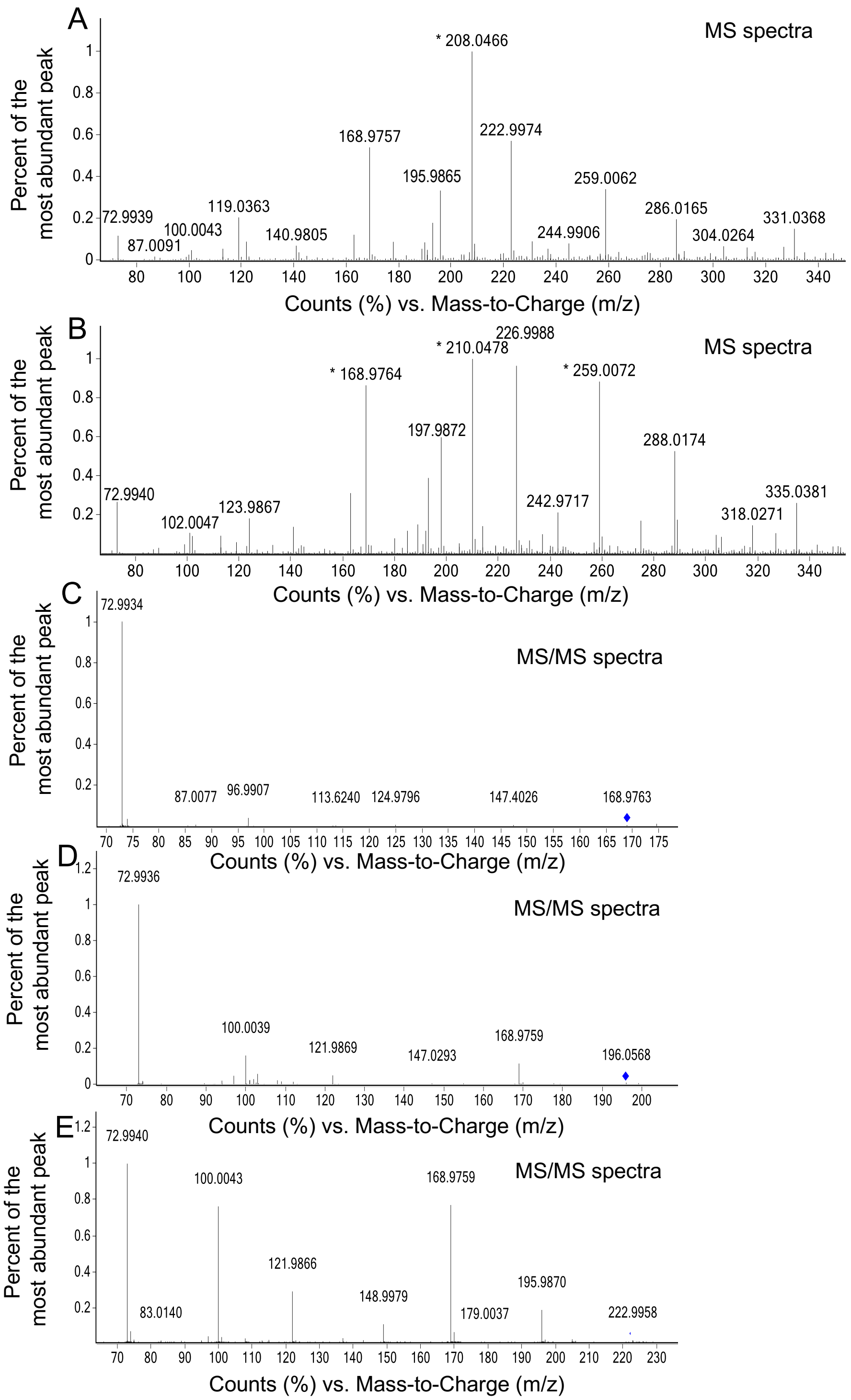

Fig. S3. Identification of the products generated from the reaction between cyanide and glyoxylate using isotope distribution comparison of $K^{12}C^{14}N$ versus $K^{13}C^{15}N$. Using tandem mass spectrometry and isotope distribution comparison, we confirmed the identity of the reaction products shown in Supplementary Figure 2A. Glyoxylate was mixed with **A**) unlabeled ($^{12}C^{14}N$) sodium cyanide or **B**) isotope ($^{13}C^{15}N$) labeled sodium cyanide. The targeted MS/MS scans of the following peaks were acquired: **C**) m/z = 168.97, **D**) m/z = 195.99, and **E**) m/z = 222.99.

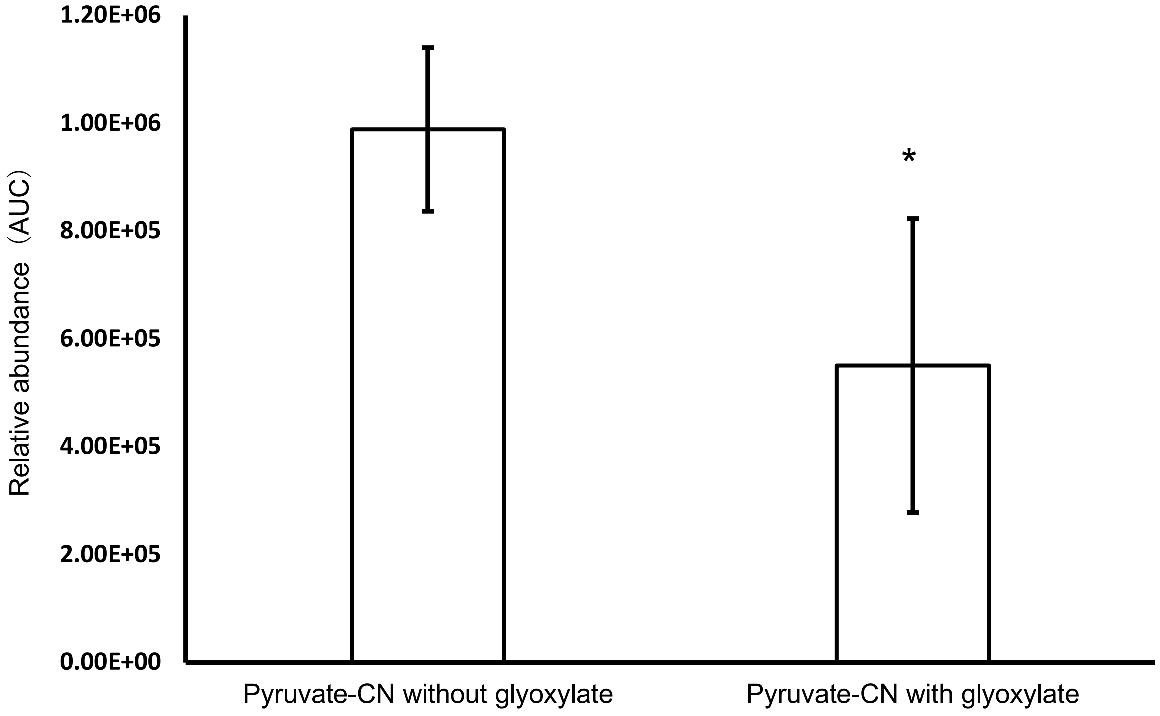

Fig. S4. Glyoxylate strips off cyanide that is bound to pyruvate. Pyruvate (100 μM) was mixed with KCN (135 mM) and incubated for 1 hour. The amount of cyanide bound to pyruvate was measured by MS in the presence or absence of glyoxylate. The area under the curve is presented. * = p=0.004

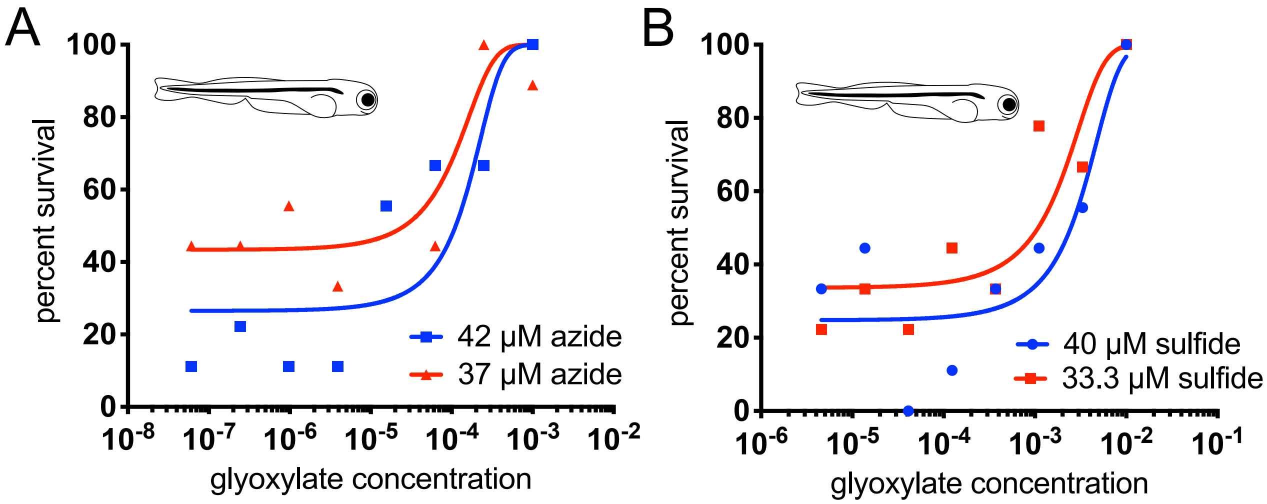

Fig S5. Glyoxylate rescues sulfide and azide lethality. (**A**) Percent survival of 5 dpf zebrafish after 20 h of exposure to given concentrations of azide or (**B**) sulfide and glyoxylate (3 fish per well with N=3 wells, data are presented as mean). EC50s for glyoxylate against these metabolic poisons were 112 μM, 26 μM, 2.5 mM, and 1.1 mM for 42 μM azide, 37 μM azide, 40 μM sulfide, and 33.3 μM sulfide respectively. 95% confidence intervals were 9.19 μM to 251.2 μM, -213.9 μM to 143.8 μM, 817.8 μM to 6.55 mM, and 143.6 μM to 3.27 mM for 42 μM azide, 37 μM azide, 40 μM sulfide, and 33.3 μM sulfide respectively. Fits shown are nonlinear regressions using the equation Y=100/(1+10^((LogEC50-X)*HillSlope))).

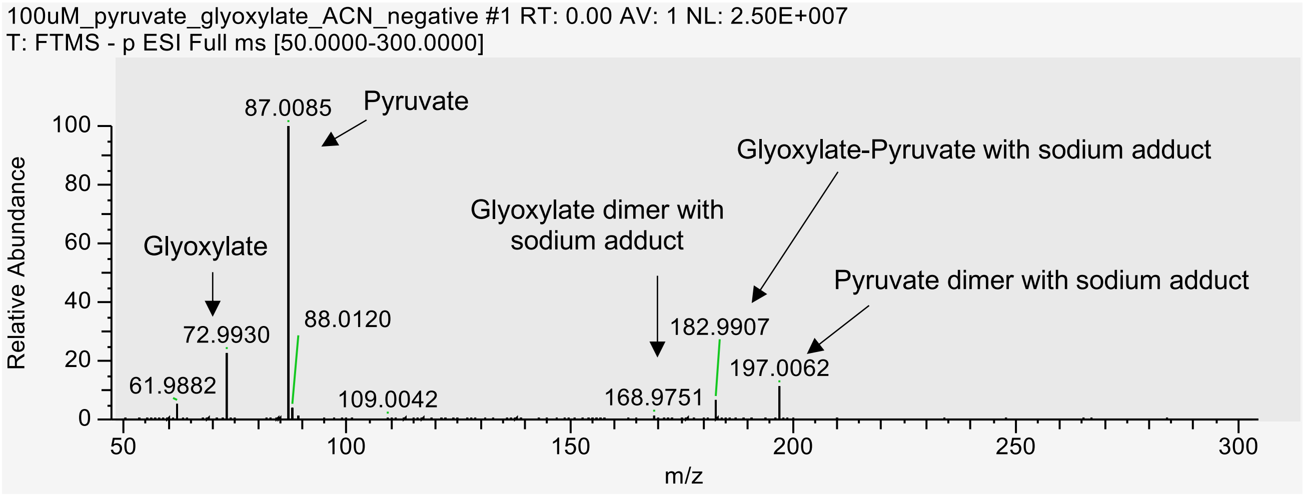

Fig. S6. Glyoxylate interacts with pyruvate to form a non-covalent complex. To determine if glyoxylate reacts with pyruvate, we used ESI-MS. We identified 3 species (m/z = 168.97, 182.99 and 197.00) demonstrating an interaction but not a reaction.

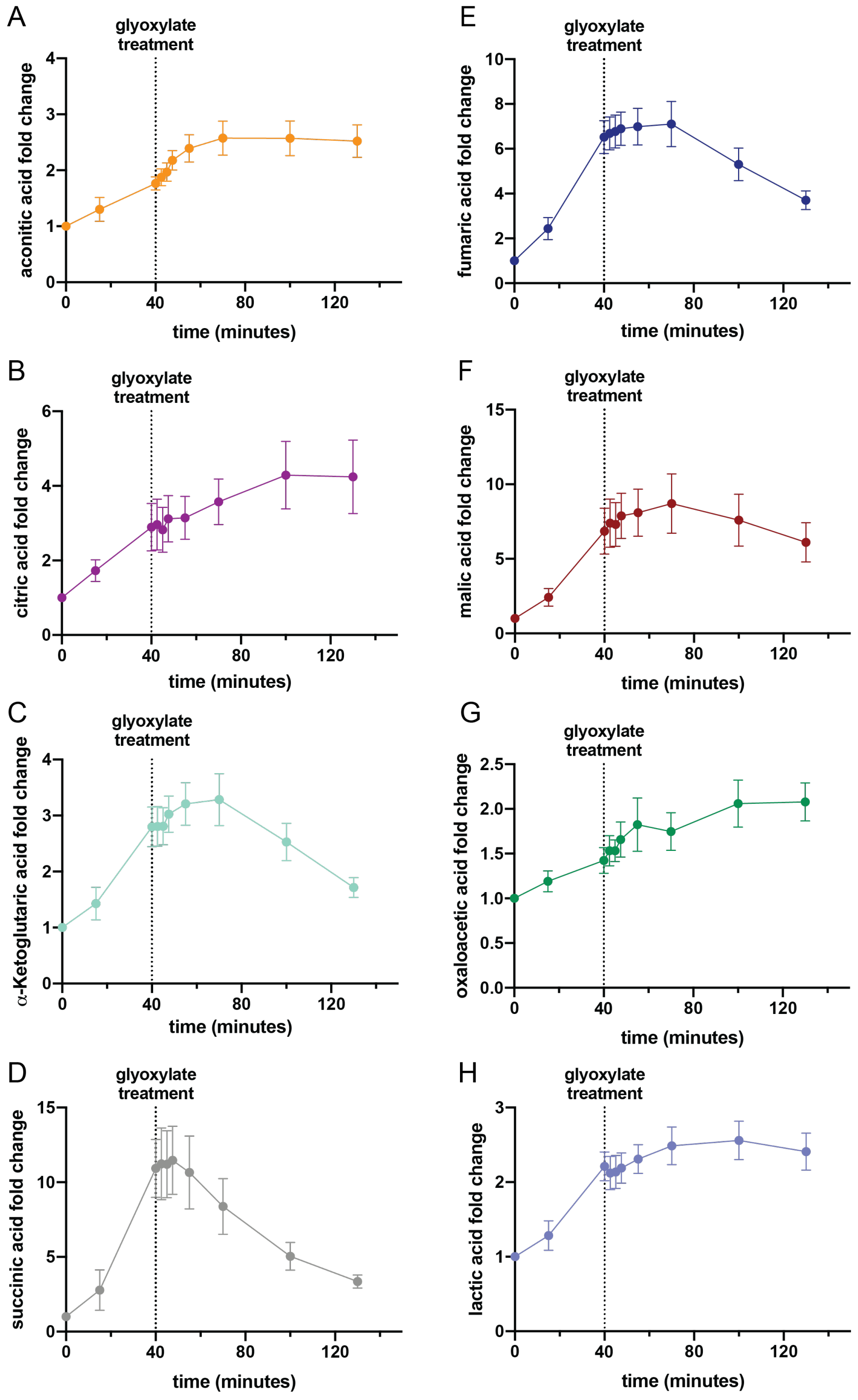

Fig. S7. Effect of glyoxylate treatment on TCA metabolites following cyanide exposure. Additional metabolite profiling performed on rabbit serial plasma samples taken before, during, and after a 40-minute cyanide infusion. Glyoxylate was administered i.m. at 40 minutes, following the cyanide infusion. Mean fold change from baseline ± SD shown for aconitic acid (A), citric acid (B), α-ketoglutaric acid (C), succinic acid (D), fumaric acid (E), malic acid (F), oxaloacetic acid (G), and lactic acid (H)

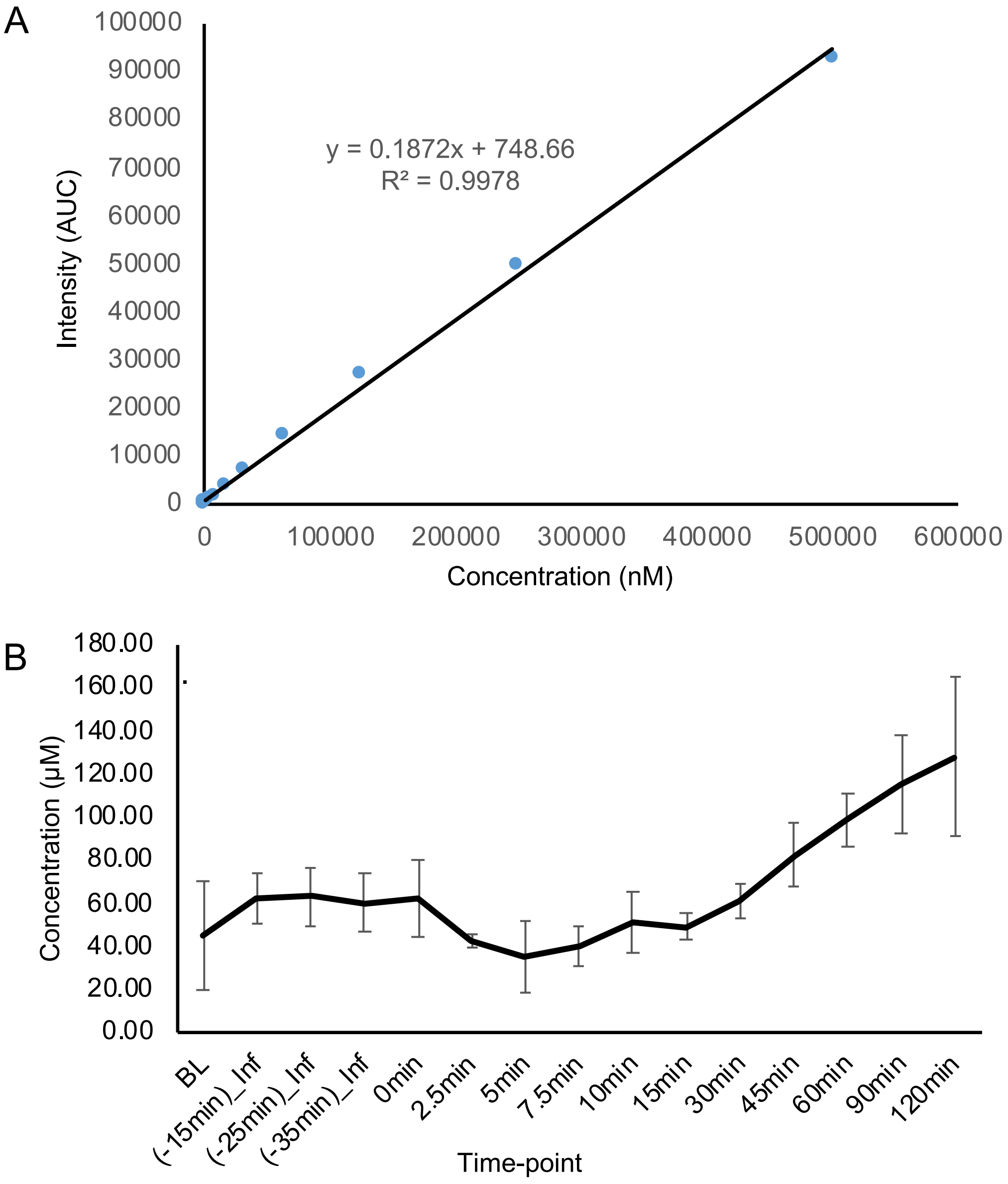

Fig. S8. Absolute quantification of pyruvate levels in rabbit serum. A) Pyruvate-^{13}C standard curve was generated in pooled reference plasma using isotopically labeled pyruvate. The linear range is 30.5 nM to 500 μM. Baseline pyruvate concentration in rabbit plasma was 44.56 ± 25.03 μM (n=3). **B)** Rabbits (n=3) were infused with glyoxylate in the absence of cyanide and the absolute plasma concentration of pyruvate was measured. The maximum fold increase was ~2.87 at 120 minutes post infusion as compared to the baseline. Data is presented as mean ± standard deviation.

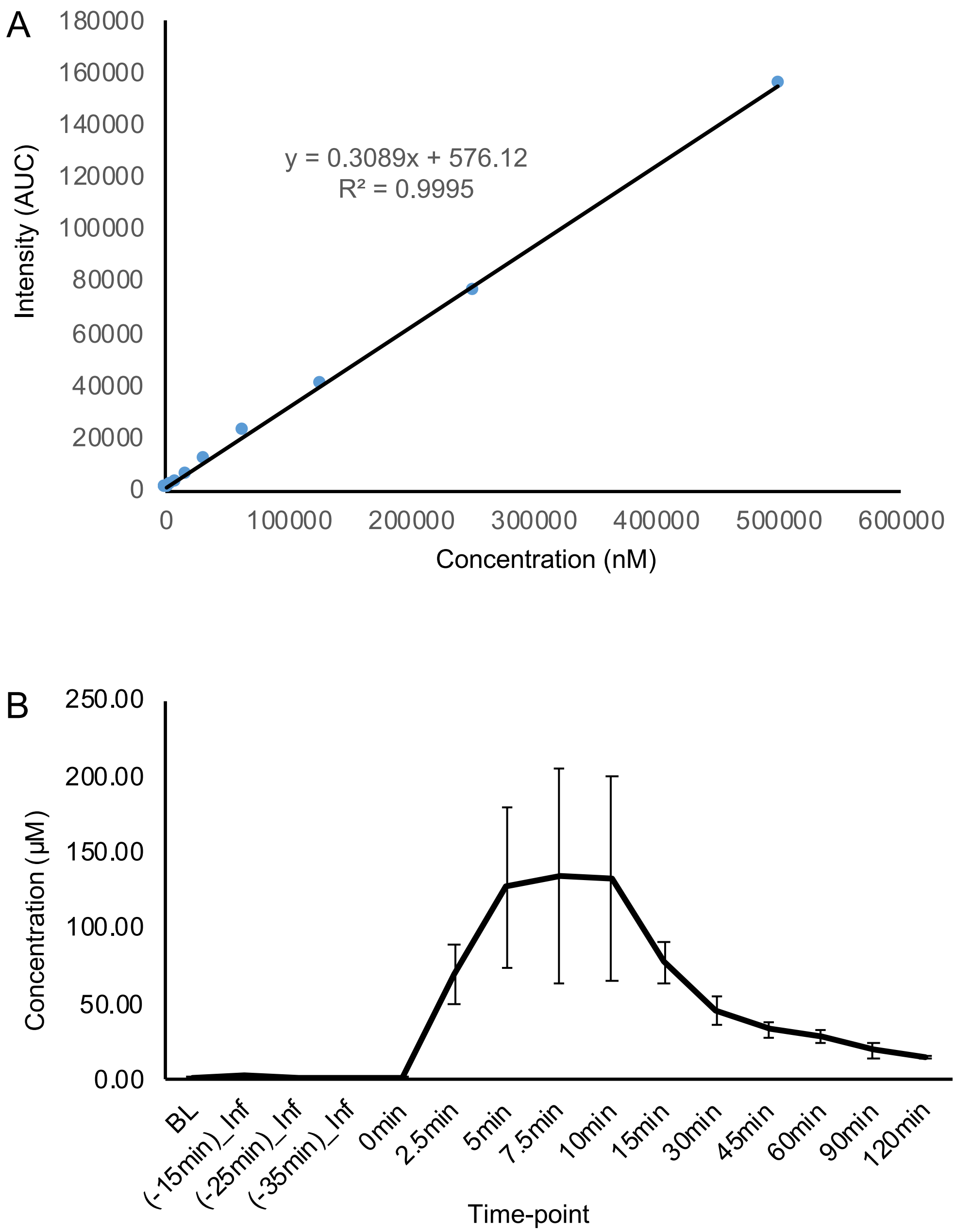

Fig. S9. Absolute quantification of glyoxylate levels in rabbit serum. A) Glyoxylate-$^{13}C_2$ standard curve was generated in pooled reference plasma using isotopically labeled glyoxylate. The linear range is 122 nM to 500 μM. The baseline glyoxylate concentration in rabbit plasma was 1.2 ± 0.36 μM (n=3). **B)** Rabbits (n=3) were infused with glyoxylate in the absence of cyanide and the absolute plasma concentration of glyoxylate was measured. The maximum fold increase was ~112 at 7.5 minutes post infusion as compared to the baseline. Data is presented as mean ± standard deviation.

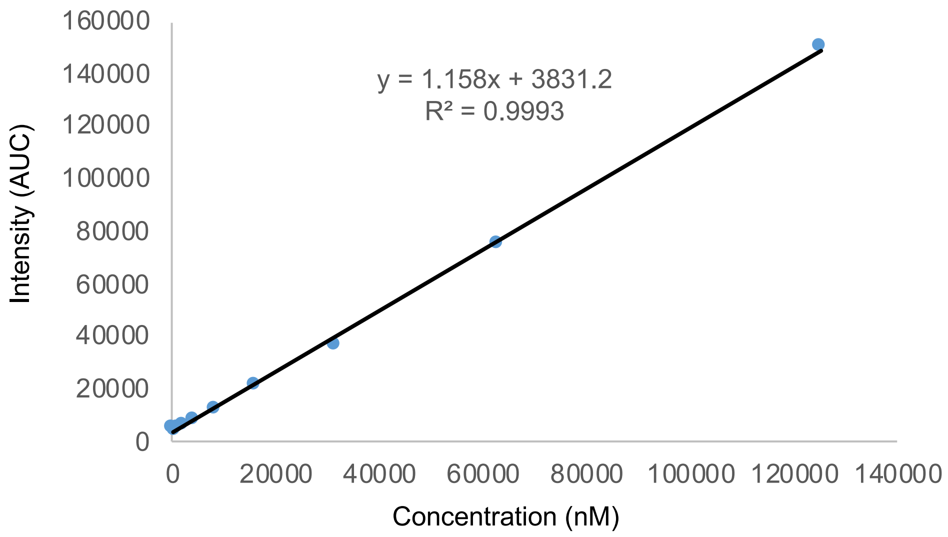

Fig. S10. Quantification of oxalate levels in rabbit serum. A) Oxalate standard curve was generated in pooled reference plasma by spiking in oxalate. The linear range is 61 nM to 125 μM. The baseline oxalate concentration in rabbit plasma was 2.30 ± 0.92 μM (n=3).

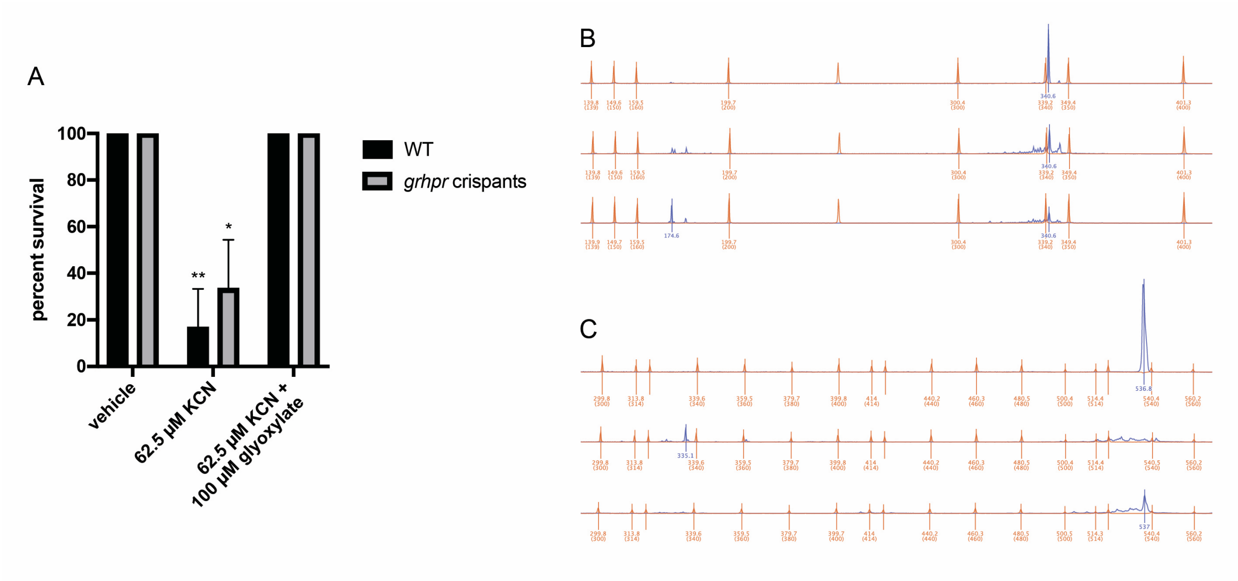

Fig. S11. Effect of *grhpr* knockdown on glyoxylate rescue. (A) Percent survival of 5 dpf wild type TuAB zebrafish vs F0 *grhpr* crispants 20 h after treatment with either cyanide, cyanide and glyoxylate, or water. Mean ± SEM shown. There was a significant difference in survival between vehicle vs KCN treated WT fish and a significant difference in survival between vehicle-treated WT fish vs *grhpr* crispants treated with KCN (* $p<0.05$; ** $p<0.01$ using one-way ANOVA). 3 fish per well, N=3 wells each for vehicle treatment in WT fish and *grhpr* crispants, N=6 wells each for KCN treatment in WT fish and *grhpr* crispants, N=6 wells each for KCN + glyoxylate treatment in WT fish and *grhpr* crispants. **(B)** Representative fragment analysis of 6-FAM labelled PCR product of *grhpra* CRISPR-targeted region in 3 WT fish (top row) vs 3 crispant fish in each of the bottom two traces. **(C)** Representative fragment analysis of 6-FAM labelled PCR product of *grhprb* CRISPR-targeted region in 3 WT fish (top row) vs 3 crispant fish in each of the bottom two traces.

Table S3. CRISPR guide and fragment analysis primer sequences for *grhpr*

Target	gRNA targeting sequence	Forward primer	Reverse primer	Product Size
grhpra exon 3	AGCGAGGTGGTCAAATCCCA	TCCCTATTAAACACATCCAGGG	AGGCATCGACGACTGACTTT	340 bp
grhpra exon 4	GGTCTCAGAGGAATACGAGT			
grhprb exon 2	TGATGACCCCGTGCCCCGAG	TGTGTTGAGGCAAGATGGAG	CAATTTTGGGTGAACCATCC	540 bp
grhprb exon 2	AGCAGCTCCACTCGGGGCAC			
grhprb exon 3	GTCCTCAGCACCATGTCAGT			

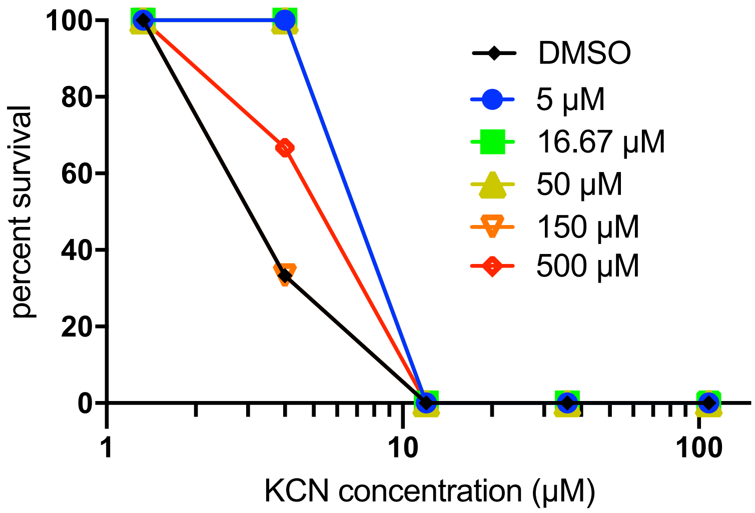

Fig. S12. Effect of LDH inhibition on cyanide toxicity. 5 dpf zebrafish were arrayed in a 96 well plate, 3 per well and were preincubated for 15 min with the given concentrations of the LDH inhibitor, GSK2837808A, or DMSO alone such that there was a final concentration of 1% DMSO in each well. Fish were then exposed to varying doses of KCN for 24 h and then survival was assessed. No siginficant differences were found between any of the groups by one way ANOVA.

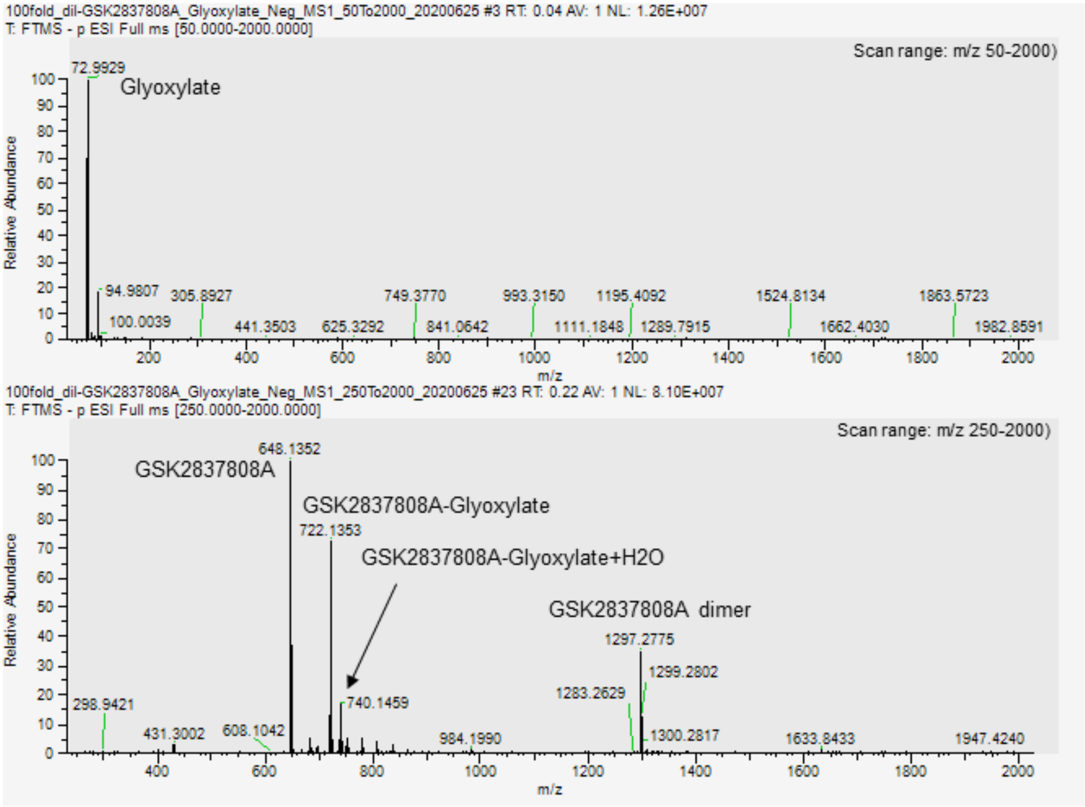

Fig. S13. Glyoxylate can interact with GSK2837808A to form a non-covalent complex. To determine if glyoxylate reacts with GSK2837808A, we used ESI-MS. We identified 3 species (m/z = 648.13, 740.14 and 1297.27).
